# Integrated metabolomics and lipidomics study of patients with atopic dermatitis in response to dupilumab

**DOI:** 10.3389/fimmu.2022.1002536

**Published:** 2022-10-20

**Authors:** Lishan Zhang, Xueyi Wen, Yibo Hou, Yongshi Yang, Wei Song, Yueping Zeng, Jinlyu Sun

**Affiliations:** ^1^ Department of Allergy, State Key Laboratory of Complex Severe and Rare Diseases, Peking Union Medical College Hospital, Chinese Academy of Medical Sciences and Peking Union Medical College, Beijing, China; ^2^ Allergy Department, Beijing Key Laboratory of Precision Medicine for Diagnosis and Treatment of Allergic Diseases, National Clinical Research Center for Dermatologic and Immunologic Diseases, Peking Union Medical College Hospital, Chinese Academy of Medical Sciences and Peking Union Medical College, Beijing, China; ^3^ Medical Science Research Center, Peking Union Medical College Hospital, Chinese Academy of Medical Science and Peking Union Medical College, Beijing, China; ^4^ Department of Dermatology, State Key Laboratory of Complex Severe and Rare Diseases, Peking Union Medical College Hospital, Chinese Academy of Medical Sciences and Peking Union Medical College, National Clinical Research Center for Dermatologic and Immunologic Diseases, Beijing, China

**Keywords:** atopic dermatitis, dupilumab, metabolomics, lipidomics, different response

## Abstract

**Background:**

Atopic dermatitis (AD) is one of the most common chronic inflammatory skin diseases. Dupilumab, a monoclonal antibody that targets the interleukin (IL)-4 and IL-13 receptors, has been widely used in AD because of its efficacy. However, metabolic changes occurring in patients with AD in response to dupilumab remains unknown. In this study, we integrated metabolomics and lipidomics analyses with clinical data to explore potential metabolic alterations associated with dupilumab therapeutic efficacy. In addition, we investigated whether the development of treatment side effects was linked to the dysregulation of metabolic pathways.

**Methods:**

A total of 33 patients with AD were included in the current study, with serum samples collected before and after treatment with dupilumab. Comprehensive metabolomic and lipidomic analyses have previously been developed to identify serum metabolites (including lipids) that vary among treatment groups. An orthogonal partial least squares discriminant analysis model was established to screen for differential metabolites and metabolites with variable importance in projection > 1 and p < 0.05 were considered potential metabolic biomarkers. MetaboAnalyst 5.0 was used to identify related metabolic pathways. Patients were further classified into two groups, well responders (n = 19) and poor responders (n = 14), to identify differential metabolites between the two groups.

**Results:**

The results revealed significant changes in serum metabolites before and after 16 weeks of dupilumab treatment. Variations in the metabolic profile were more significant in the well-responder group than in the poor-responder group. Pathway enrichment analysis revealed that differential metabolites derived from the well-responder group were mainly involved in glycerophospholipid metabolism, valine, leucine and isoleucine biosynthesis, the citrate cycle, arachidonic acid metabolism, pyrimidine metabolism, and sphingolipid metabolism.

**Conclusion:**

Serum metabolic profiles of patients with AD varied significantly after treatment with dupilumab. Differential metabolites and their related metabolic pathways may provide clues for understanding the effects of dupilumab on patient metabolism.

## Introduction

Atopic dermatitis (AD) is a chronic, pruritic, immune-mediated inflammatory skin disease characterized by a predominant type 2 immune response (1). AD has the highest prevalence among skin diseases, and is estimated to affect approximately 20% of children and 2.1–4.9% of adults (2–4). The pathogenesis of AD is complex and involves genetic susceptibility, impaired skin barrier function, immune dysfunction, and environmental and psychological factors (5). Although the precise causes of AD are not fully understood, evidence has shown that T helper (Th)2 cells cytokines such as interleukin (IL)-4 and IL-13 are important cytokines involved in the development of AD. In recent years, Th1/Th17/Th22 were also reported to play important roles in AD.

Metabolomics is a relatively new and developing technology in biological sciences that offers abundant information relevant to biomarkers discovery, disease pathogenesis, mechanisms of drug action, and precise therapeutics *via* the identification and measurement of small-molecule metabolites (6). The primary analytical tools used in metabolomics include nuclear magnetic resonance, liquid chromatography-mass spectrometry (LC-MS), and gas chromatography-MS (GC-MS) (7). Lipidomics is considered a subset of metabolomics that focuses on the study of the lipidome (8). Abnormalities in lipid metabolism are strongly related to the development of AD (9). However, it is not yet possible to detect all metabolites with a single technology; thus, different detection platforms are required to provide a comprehensive metabolic profile. In the last decade, only a few studies have directly addressed AD metabolomics or lipidomics (10–15). These studies have shown that serum from patients with AD have significantly different metabolite profiles compared to healthy individuals.

However, knowledge regarding the metabolic changes observed following AD treatment is limited. Hotze et al. demonstrated that patients with AD who responded well to omalizumab had altered lipid metabolites profiles with high levels of several glycerophospholipids (GP), specifically phosphatidylcholine (PC) (12). Dupilumab, a humanized IgG4 monoclonal antibody that binds to the IL-4 receptor alpha chain, has been widely used due to its efficacy and safety as a treatment for AD. To the best of our knowledge, metabolite biomarkers associated with dupilumab treatment response and changes in relevant metabolic pathways have not been elucidated.

In this study, LC-MS and GC-MS were applied to investigate comprehensive metabolomic and lipidomic alterations in patients with AD before and after treatment with dupilumab. Additionally, we identified differentially expressed metabolites in patients with positive treatment and used Kyoto Encyclopedia of Genes and Genomes (KEGG) database to analyze their associated metabolic pathways, which may add to our understanding of the metabolic mechanisms influenced by dupilumab treatment.

## Methods

### Study population

We recruited 33 patients diagnosed with moderate to severe AD (16) at the dermatology outpatient clinic of the Peking Union Medical College Hospital from March 2021 to February 2022. **Table S1** lists the inclusion and exclusion criteria of the study population (**Supplementary Table S1**). Eligible patients were treated with dupilumab (loading dose of 600mg subcutaneously, and 300mg every 2 weeks). AD severity was assessed using the Eczema Area and Severity Index (EASI) and the scoring atopic dermatitis (SCORAD) score. All the enrolled participants were treated with dupilumab for 16 weeks. At the end of follow-up, patients were grouped as well or poor responders to the medication, based on whether they reached EASI-75% (≥ 75% improvement from baseline) (**Figure S1**). All scores were qualitatively analyzed by two researchers blinded to each other’s decisions (LS Zhang and XY Wen). The final grading decision was made by a senior grader (YP Zeng). The current study was approved by the Regional Ethical Review Board of the Peking Union Medical College Hospital (ZS-3159). Written informed consent was obtained from all participants prior to their enrollment in the present study.

### Serum sample preparation

Serum samples from each participant were obtained after overnight fasting at the outpatient clinic of the Peking Union Medical College Hospital and were collected before and after 16 weeks of dupilumab treatment. The serum samples were immediately frozen at -80°C until analysis.

### Measurement of serum total IgE levels and absolute eosinophil count

Pre- and post-therapeutic levels of serum total immunoglobulin E (tIgE) and absolute eosinophil count (AEC) were assessed. tIgE levels were measured using ImmunoCAP (Phadia, Uppsala, Sweden), and AEC results were obtained from routine blood tests.

### Measurement of serum cytokines

The serum concentrations of IL-2, IL-4, IL-6, IL-10, tumor necrosis factor-α (TNF-α), interferon-γ (IFN-γ), and IL-17A before and after dupilumab treatment were measured by a cytometric bead array (CBA) Human TH1/TH2/TH17 cytokine kit (Catalog No. 560484, BD Biosciences). Samples were processed and analyzed according to the manufacturer’s recommended protocol.

### GC-TOF-MS analysis

Sample preparation and extraction steps were shown in **Supplementary Data 1**. GC-TOF-MS analysis was performed using an Agilent 7890 gas chromatograph coupled with TOF-MS (see **Supplementary Data 1** for the specific analysis conditions of GC-TOF-MS).

### LC-MS analysis

Metabolites and lipids were extracted from the samples (See **Supplementary Data 1** for further details of methodology). For metabolomics studies, an ultraperformance LC (UPLC) Vanquish system (Thermo Fisher Scientific, Waltham, MA, USA) with a UPLC BEH Amide column (2.1 mm × 100 mm, 1.7 μm) coupled to a Q Exactive HFX mass spectrometer (Orbitrap MS, Thermo Fisher) was used for separation and subsequent analysis (see **Supplementary Data 1** for more details). For lipidomics, a 1290 UPLC system (Agilent Technologies, Santa Clara, CA, USA), equipped with a Kinetex C18 column (2.1 x 100 mm, 1.7 μm, Phenomen) coupled to a Q Exactive HFX mass spectrometer (Orbitrap MS, Thermo Fisher), was used for separation and later analysis (see **Supplementary Data 1** for more details).

### Data processing and statistical analysis

SPSS software (version 20.0) was used to perform a Student’s *t*-test and the Mann-Whitney U test when comparing continuous variables between treatment response patient groups. For categorical variables, the chi-square test or Fisher’s exact test was used. Results were expressed as mean ± SD with p < 0.05 considered statistically significant. Relationship between two parametric were evaluated with Pearson’s rank correlation.

Raw data obtained from both metabolomics and lipidomics analyses were processed using the XCMS software (17), including peak detection, extraction, alignment, and integration. The data are available at the NIH Common Fund’s National Metabolomics Data Repository (NMDR) website, the Metabolomics Workbench, https://www.metabolomicsworkbench.org where it has been assigned Project IDST002302. The data can be accessed directly *via* it’s Project DOI: 10.21228/M82m60. This work is supported by NIH grant U2C-DK119886 (18). Metabolite annotation was based on the retention index values and MS spectra from the in-house MS/MS database established by Biotree Biotech Co. (Shanghai, China) and the LECO-Fiehn Rtx5 database. Lipid identification was performed using the LipidBLAST library. The cut-off for annotation was set at 0.3. When a metabolite was detected by both the LC-MS and GC-MS platforms, the signal detected by GC-MS was used for statistical analyses. If the same metabolite was detected in both the negative and positive ion modes, the signal with the highest intensity was used for statistical analysis.

Data acquired from metabolomics and lipidomics analyses were normalized and subjected to multivariate statistical analysis using simca 14.0 software, which included principal component analysis (PCA) and supervised orthogonal partial least squares discriminant analysis (OPLS-DA). Cross-validation of OPLS-DA models obtained from 200 permutation tests. The quality of the OPLS-DA model was assessed using R^2^ and Q^2^ values. Differential metabolites were screened based on variable importance in projection (VIP) > 1 and p < 0.05. VIP values were derived from the OPLS-DA model and p-values from a two-tailed Student’s *t*-test on normalized peak areas between the two groups. A heatmap was plotted using a free online platform for data analysis and visualization (https://www.bioinformatics.com.cn). Metabolic pathway analysis of differentially expressed metabolites was performed using MetaboAnalyst 5.0 (https://www.metaboanalyst.ca/).

## Results

### Study population characteristics

A total of 33 patients with AD, who had completed a 16-week dupilumab treatment course and all follow-ups, were divided into well- and poor-responder groups according to improvements in EASI. Overall, 58% of patients achieved EASI-75% at week 16 compared to baseline and were placed in the well-responder group. No significant differences were found in age, sex ratio, or body mass index between the two groups. tIgE levels were lower in the well-responder group than in the poor-responder group; however, this difference was not statistically significant. We also observed that the incidence of adverse events in the well-responder group was significantly higher than in the poor-responder group. The demographic and clinical characteristics of the 33 patients with AD included in the study are shown in **Table 1**.

**Table 1:** Characteristic of the study population.

	Total (n = 33)	Well-responder (n = 19)	Poor-responder (n = 14)	*p-values
Age	32.54 ± 11.75	32.53 ± 10.06	32.57 ± 14.12	0.08
Sex, (male), n (%)	17 (51.5%)	8 (42.1%)	9 (64.3%)	0.70
BMI, kg/m^2^	24.21 ± 4.20	23.37 ± 4.24	25.34 ± 4.0	0.91
Atopic comorbidities				
Asthma, n (%)	7	3	3	–
Allergic rhinitis,n (%)	21	13	8	–
Food allergy, n (%)	2	1	1	–
Previous systemic therapy				
Only Antihistamines	7	2	5	–
Ciclosporin	15	12	3	–
Oral corticosteroids	5	4	1	–
SCORAD score at baseline	62.07 ± 13.64	62.85 ± 17.30	61.01 ± 6.42	–
EASI score at baseline	31.87 ± 14.85	32.92 ± 18.63	30.46 ± 7.67	–
Serum tIgE at inclusion [IU/ml]	1966.71 ± 1948.58	1787.01 ± 1899.23	2210.57 ± 2059.30	0.28
Eosinophil count (10^9^/L) at baseline	0.51 ± 0.59	0.59 ± 0.76	0.38 ± 0.20	0.24
SCORAD score at visit 16w	27.61 ± 10.87	22.76 ± 6.64	34.20 ± 12.20	–
EASI score at visit 16w	9.44 ± 6.79	6.21 ± 4.06	13.82 ± 7.40	–
Serum tIgE at visit 16w [IU/ml]	1331.42 ± 1838.00	1303.19 ± 1807.94	1369.73 ± 1946.07	0.69
Eosinophil count (10^9^/L) at visit 16w	0.41 ± 0.32	0.43 ± 0.38	0.39 ± 0.23	0.31
Total adverse events at week 16	11(33.3%)	8(42.1%)	3(21.4%)	0.00

Except where indicated otherwise, values are the mean ± SD. SCORAD, scoring atopic dermatitis, EASI, eczema area and severity index, tIgE, total immunoglobulin E. *p values, well-responder vs poor-responder.

### Effect of dupilumab on levels of serum cytokines of patients with AD

Analysis of serum cytokines showed that IL-6, IL-10, and IFN-γ were significantly decreased in patients after dupilumab treatment (P<0.05). However, no statistical differences were observed for IL-2, IL-4, TNF-α, and IL-17A (**Figure S2**).

### Integrative metabolomic analysis reveals the metabolic profile of AD patients before and after dupilumab treatment

**Figure 1:**
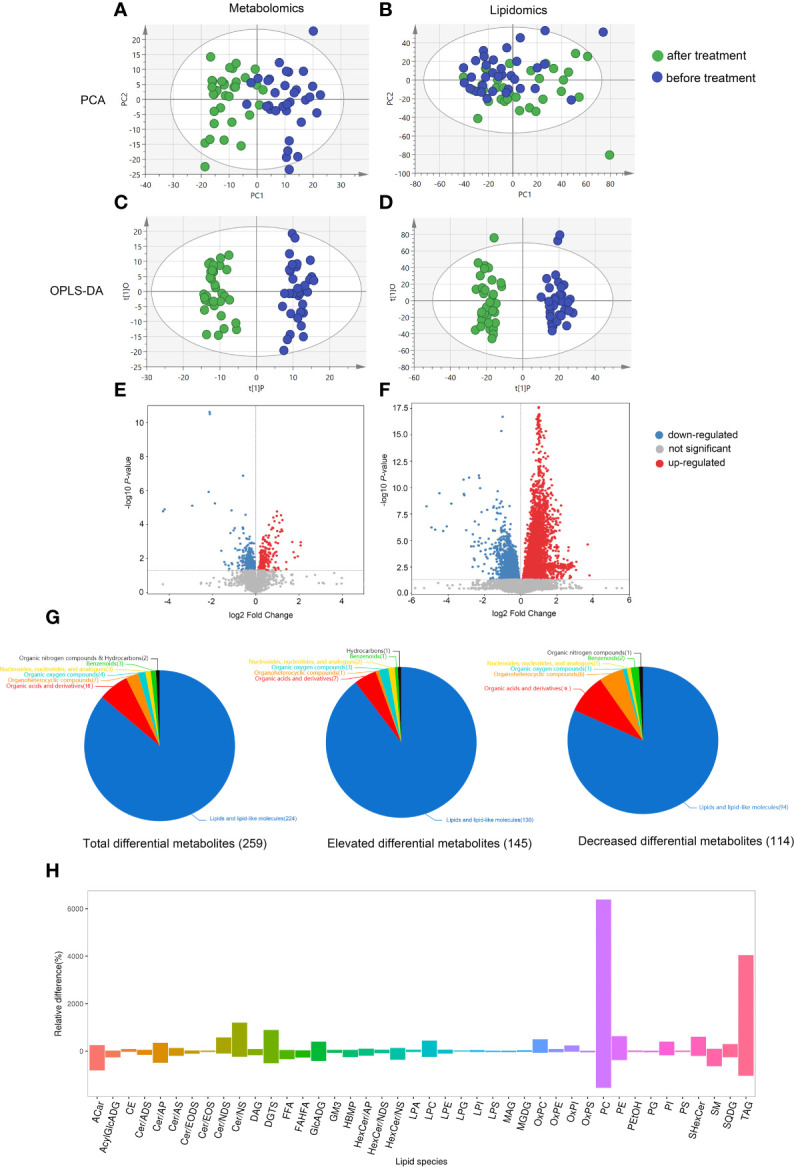
Metabolomic and lipidomic profiles. **(A–D)** Principal component analysis (PCA) and orthogonal partial least squares discriminant analysis (OPLS-DA) score plots of the metabolomics and lipidomics data. After-treatment group (green), before-treatment group (blue). **(E, F)** Volcano diagrams showing all of the identified metabolites from metabolomics and lipidomics analysis. The x-axis and y-axis were drawn based on the fold change and p-values. Each dot represents a metabolite. The larger dots indicate higher VIP value. The increased and decreased metabolites in the after-treatment group are represented by the red and blue dots, respectively, whereas the gray dots represent the unchanged metabolites between two group. **(G)** Pie charts show total, elevated and decreased differential metabolites. Different colors indicate different classes. **(H)** Lipidomics bar plot showing the degree of variation in the content of different types of lipids. The y-axis represents the relative percentage change. If the relative percentage change is positive, it means that this super class of lipid is higher in after-treatment group. The x-axis represents the classification of lipids. Acar, Acylcarnitine; AcylGlcADG, Acylglucuronosyldiacylglycerol; CE, Cholesteryl ester; Cer/ADS, Ceramide alpha-hydroxy fatty acid-dihydrosphingosine; Cer/AP, Ceramide alpha-hydroxy fatty acid-phytospingosine; Cer/AS, Ceramide alpha-hydroxy fatty acid-sphingosine; Cer/EODS, Ceramide Esterified omega-hydroxy fatty acid-dihydrosphingosine; Cer/EOS, Ceramide Esterified omega-hydroxy fatty acid-sphingosine; Cer/NDS, Ceramide non-hydroxyfatty acid-dihydrosphingosine; Cer/NS, Ceramide non-hydroxyfatty acid-sphingosine; DAG, Diacylglycerol; DGTS, Diacylglyceryl trimethylhomoserine; FFA, Free fatty acid; FAHFA, Fatty acid ester of hydroxyl fatty acid; GlcADG, glucuronosyldiacylglycerol; GM3, Ganglioside; HBMP, Hemibismonoacylglycerophosphate; HexCer/AP, Hexosylceramide alpha-hydroxy fatty acid-phytospingosine; HexCer/NDS, Hexosylceramide non-hydroxyfatty acid-dihydrosphingosine; HexCer/NS, Hexosylceramide non-hydroxyfatty acid-sphingosine; LPA, Lysophosphatidic acid; LPC, Lysophophatidylcholine; LPE, Lysophosphatidylethanolamine; LPG, Lysophosphatidylglycerol; LPI, Lysophosphatidylinositol; LPS, Lysophosphatidylserine; MAG, Monoacylglycerol; MGDG, Monogalactosyldiacylglycerol;OxPC, Oxidized phosphatidylcholine; OxPE, Oxidized phosphatidylethanolamine; OxPI, Oxidized phosphatidylglycerol; OxPS, Oxidized phosphatidylinositol; PC, Phosphatidylcholine; PE, Phosphatidylethanolamine; PEtOH, Phosphatidylethanol; PG, Phosphatidylglycerol; PI, Phosphatidylinositol; PS, Phosphatidylserine; SHexCer, SulfurHexosylceramide hydroxyfatty acid; SM, Sphingomyelin; SQDG, Sulfoquinovosyl diacylglycerol; TAG, triacylglycerol.

We investigated the longitudinal effects of dupilumab treatment on the serum metabolic profiles of patients with AD. In the present study, we identified 849 metabolites and 1753 lipid species in patient serum. PCA, a multivariate technique was used to determine whether samples from different groups could be segregated based on their metabolic profiles. The PCA results showed that a large difference between the after- and before-treatment groups in the metabolomics dataset; however, the PCA did not show an obvious separation between the groups in the lipidomics dataset (**Figures 1A, B**). An OPLS-DA model was used to identify the greatest discriminant between the two groups. The model parameters were R^2^Y = 0.958 and Q^2^ = 0.744 in the metabolomics analysis and R^2^Y = 0.961 and Q^2^ = 0.761 in the lipidomics analysis, suggesting that the models were reliable and did not suffer from overfitting (**Figure S3**). The OPLS-DA score plot showed a clear difference in both the metabolic and lipidomic profiles (**Figures 1C, D**).

Univariate analyses, including the t-test and fold change analysis, were used to detect changes in metabolite levels between the before and after treatment groups. Multivariate statistical analyses were also used to screen for differential metabolites (VIP scores > 1, p < 0.05). Forty-one metabolites and 218 lipids met these criteria (**Table S2**). A volcano map was drawn to visually display the overall distribution of metabolic differences between the groups (**Figures 1E, F**). We then assigned the total differential metabolites to seven categories according to the Human Metabolome Database (HMDB). The differences between the groups were largely attributed to lipid and lipid-like molecule contribution, with 145 elevated and 114 decreased, indicating that lipid metabolism in AD patients was greatly influenced by dupilumab treatment (**Figure 1G**). The lipidomics bar plot further shows clear changes in the different classes of lipids. For example, the levels of phosphatidylcholine (PC) and triacylglycerol (TAG) were significantly higher in the post-treatment group (**Figure 1H**).

**Figure 2:**
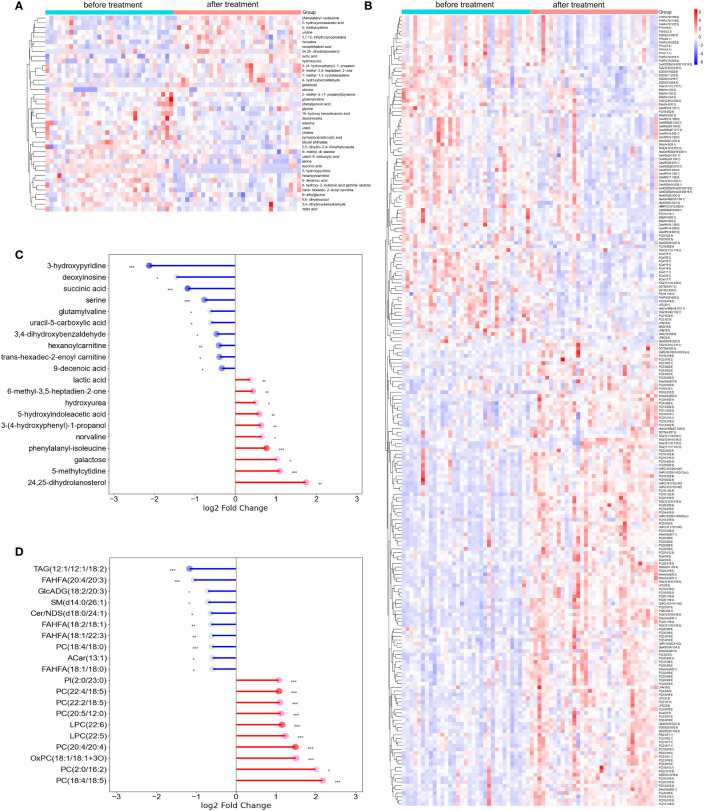
The representative differential metabolites identified from metabolomics and lipidomics analysis. **(A, B)** Hierarchical clustering analysis of metabolome and lipid profiles. Red means levels of metabolites increased after dupilumab therapy, while blue is decreasing. **(C, D)** The matchstick diagram showed the top 20 significantly change metabolites in metabolomics and lipidomics analysis based on fold change. The color depth of the dot represents the VIP value. Acar, Acylcarnitine; Cer/ADS, Ceramide alpha-hydroxy fatty acid-dihydrosphingosine; Cer/AP, Ceramide alpha-hydroxy fatty acid-phytospingosine; Cer/AS, Ceramide alpha-hydroxy fatty acid-sphingosine; Cer/EODS, Ceramide Esterified omega-hydroxy fatty acid-dihydrosphingosine; Cer/NDS, Ceramide non-hydroxyfatty acid-dihydrosphingosine; Cer/NS, Ceramide non-hydroxyfatty acid-sphingosine; DAG, Diacylglycerol; DGTS, Diacylglyceryl trimethylhomoserine; FFA, Free fatty acid; FAHFA, Fatty acid ester of hydroxyl fatty acid; GlcADG, glucuronosyldiacylglycerol; HBMP, Hemibismonoacylglycerophosphate; HexCer/NDS, Hexosylceramide non-hydroxyfatty acid-dihydrosphingosine; HexCer/NS, Hexosylceramide non-hydroxyfatty acid-sphingosine; LPA, Lysophosphatidic acid; LPC, Lysophophatidylcholine; LPE, Lysophosphatidylethanolamine; MAG, Monoacylglycerol; OxPC, Oxidized phosphatidylcholine; OxPE, Oxidized phosphatidylethanolamine; OxPI, Oxidized phosphatidylglycerol; PC, Phosphatidylcholine; PE, Phosphatidylethanolamine; PG, Phosphatidylglycerol; PI, Phosphatidylinositol; SHexCer, SulfurHexosylceramide hydroxyfatty acid; SM, Sphingomyelin; SQDG, Sulfoquinovosyl diacylglycerol; TAG, triacylglycerol. * represents P<0.05, ** represents P<0.01, *** represents P<0.001.

Hierarchical clustering heatmaps were constructed to intuitively display the overall distribution of metabolic differences between groups (**Figures 2A, B**). Furthermore, a matchstick diagram showed the fold changes in the expression of differential metabolites between the two groups (**Figures 2C, D**). These results suggest that the metabolomic and lipidomic landscapes change significantly after dupilumab treatment in patients with AD.

### Correlation between serum metabolites/lipids and clinical parameters and cytokines

We investigated the association between the top 50 differential metabolites/lipids and clinical indicators of AD severity before treatment (**Figure S4**). Although the results identified the relationship between several significant metabolites/lipids and clinical parameters, the correlation coefficients were low. For example, serum levels of uracil-5-carboxylic acid were negatively correlated with EASI (r=0.46) and SCORAD (r=0.32).

Next, we analyzed the relationship between serum metabolites/lipids and the concentration of cytokines, pre- and post-dupilumab therapy. Of note, a moderate positive correlation was observed between IL-6 and glutamylvaline in pre-therapy samples (r=0.69, p<0.001) in metabolomics. As for lipidomics, IL-4 was positively associated with PC (16:0/20:5) (r=0.63, p<0.001) and IL-10 was positively correlated with PC(18:4/18:5) (r=0.61, p<0.001). Moreover, only weak correlation was found between the cytokine profiles and metabolites/lipids in post-therapy samples (**Figure S5**).

### Metabolic profiles of well- and poor-responder groups

**Figure 3:**
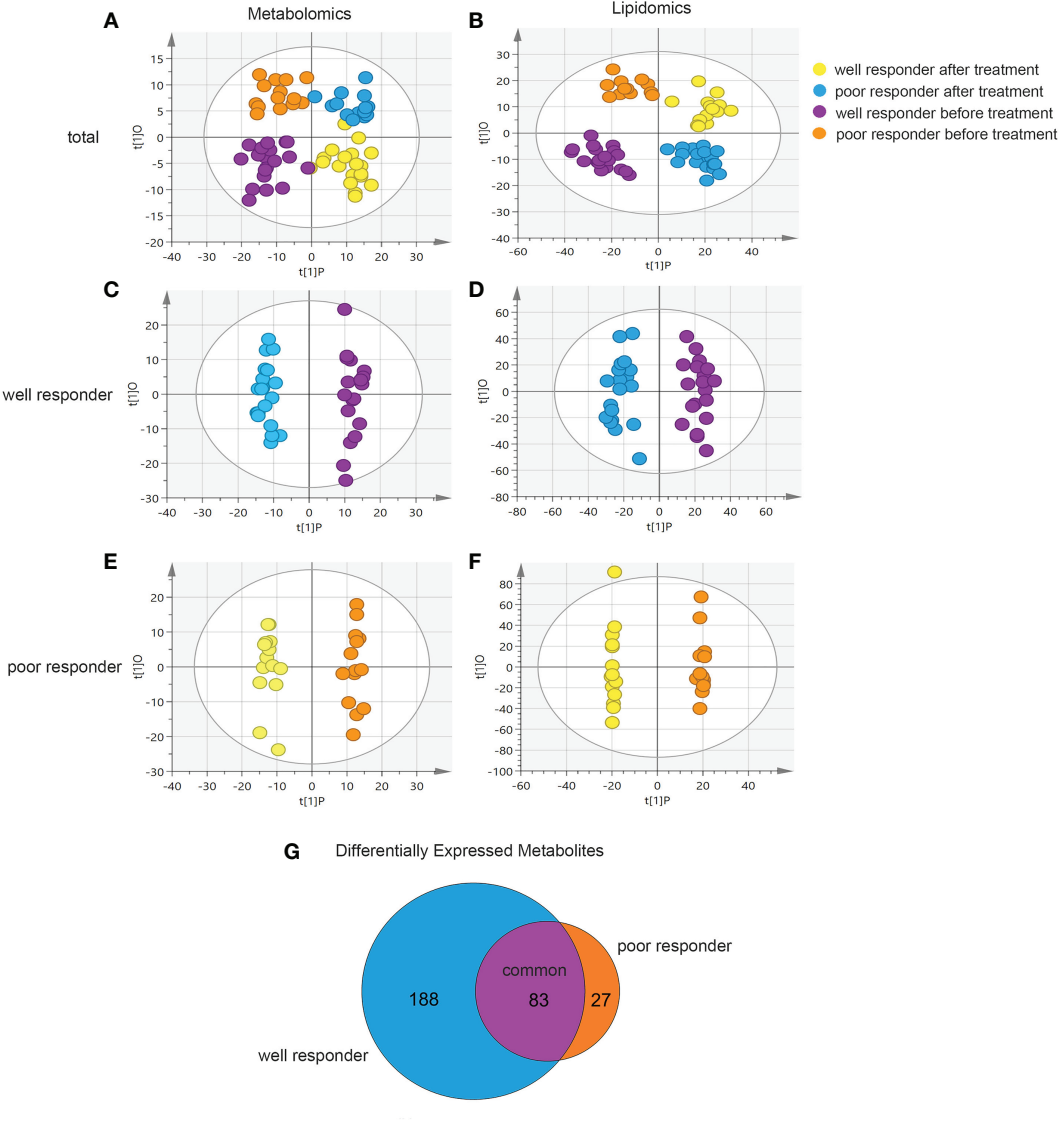
Differential between well responder and poor responder to dupilumab treatment based on metabolomics and lipidomics. **(A, B)** Orthogonal partial least squares discriminant analysis (OPLS-DA) model comparing metabolomics and lipidomics fingerprints of samples belonging to different group (well responder before treatment, poor responder after treatment, well responder before treatment and poor responder before treatment). The model parameters were R^2^Y = 0.558 and Q^2^ = 0.227 in metabolomics analysis and R^2^Y = 0.609 and Q^2^ = 0.305 in lipidomics analysis. **(C, D)** OPLS-DA model for well responder group. The model parameters were R^2^Y = 0.977 and Q^2^ = 0.736 in metabolomics analysis and R^2^Y = 0.948 and Q^2^ = 0.754 in lipidomics analysis. **(E, F)** OPLS-DA model for poor responder group. The model parameters were R^2^Y = 0.851 and Q^2^ = 0.724 in metabolomics analysis and R^2^Y = 0.998 and Q^2^ = 0.721 in lipidomics analysis. **(G)** Venn diagram analysis of differential metabolites showing shared and exclusively in the well responder or poor responder groups.

To determine whether patient metabolomes and lipidomes differ in association with their treatment response, we divided patients into well- and poor-responder groups and observed their metabolic and lipidomic profiles before and after receiving dupilumab. The OPLS-DA score plot showed evident separation between the four groups (**Figures 3A, B**; **Figure S6**). OPLS-DA models were then established for pairwise comparisons to identify differential metabolites between the different groups (**Figures 3C–F**). Given a VIP > 1 and p < 0.05, the well-responder group showed 271 differential metabolites, while the poor-responder group showed 110 differential metabolites in combined results from metabolomic and lipidomic analyses (**Figure 3G**). These results indicate that patients with AD who responded well to dupilumab treatment had remarkable serum metabolomic changes.

**Figure 4:**
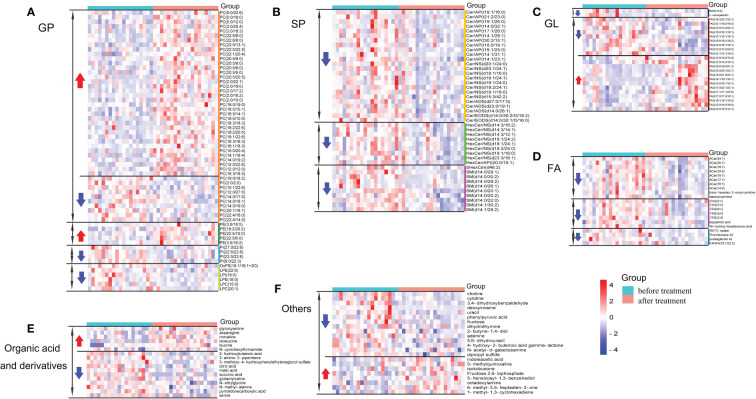
Heatmap analysis of unique differentially expressed metabolites specific for well response patients. Heatmap visualization of **(A)** altered lipids in glycerophospholipids (GP) including PC, PE, PI, Lysophospholipid (LP), and their derivative **(B)** altered lipids in sphingolipids (SP) including SM, different kinds Cer and its derivative. Besides, beta-glyrrhetinic acid and 4-androsten-11beta-ol-3,17-dione 4 belong to PR and ST, respectively **(C)** altered lipids in glycerolipids (GL), including TAG and MAG **(D)** altered lipids in fatty acid (FA) including ACar, FFA and its derivative **(E)** changes in organic acids and derivatives. **(F)** changes in some unclassified metabolites. Acar, Acylcarnitine; Cer/ADS, Ceramide alpha-hydroxy fatty acid-dihydrosphingosine; Cer/AP, Ceramide alpha-hydroxy fatty acid-phytospingosine; Cer/AS, Ceramide alpha-hydroxy fatty acid-sphingosine; Cer/EODS, Ceramide Esterified omega-hydroxy fatty acid-dihydrosphingosine; Cer/NDS, Ceramide non-hydroxyfatty acid-dihydrosphingosine; Cer/NS, Ceramide non-hydroxyfatty acid-sphingosine; FFA, Free fatty acid; FAHFA, Fatty acid ester of hydroxyl fatty acid; HexCer/AP, Hexosylceramide alpha-hydroxy fatty acid-phytospingosine; HexCer/NS, Hexosylceramide non-hydroxyfatty acid-sphingosine; LPC, Lysophophatidylcholine; LPE, Lysophosphatidylethanolamine; LPI, Lysophosphatidylinositol; MAG, Monoacylglycerol; MGDG, Monogalactosyldiacylglycerol; OxPS, Oxidized phosphatidylinositol; PC, Phosphatidylcholine; PE, Phosphatidylethanolamine; PI, Phosphatidylinositol; SHexCer, SulfurHexosylceramide hydroxyfatty acid; SM, Sphingomyelin; PR, Prenol Lipids; ST, Sterol Lipids; TAG, triacylglycerol.

To better understand the metabolic profile in AD patients who responded well to dupilumab, we conducted hierarchical clustering analysis of their unique differential metabolites. Lipids were divided into four categories based on the LIPID MAPS classification system: fatty acyls (FA), glycerolipids (GL), glycerophospholipids (GP), and sphingolipids (SP) (**Figures 4A-D**). Each of these four categories consisted of further lipid subclasses. The majority of differential metabolites were GP and SP. We found that PC was the most prominent differential lipid within the GP class, accounting for approximately 73% of all differential GP, with most showing increased levels after dupilumab treatment. The second most abundant lipid subclass, ceramide (Cer), which belongs to the SP class, was significantly decreased after dupilumab treatment. Additionally, another two clusters were created to display the change in the metabolic profile between the two groups (**Figures 4E, F**). In general, these results indicate that dupilumab treatment induced lipidomic and metabolomic changes in AD patients that exhibited a good response to therapy.

### Metabolic pathway analysis

**Figure 5:**
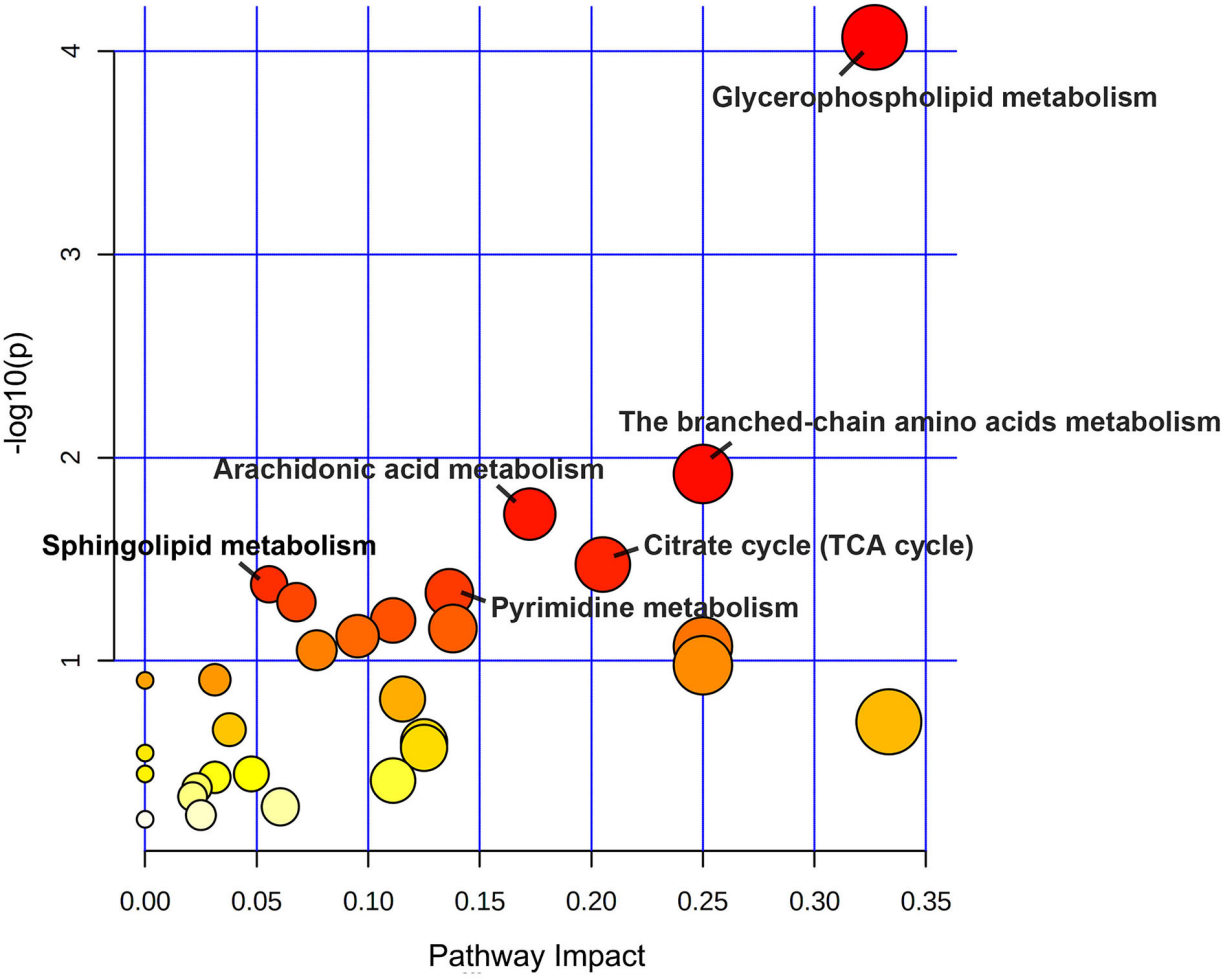
The KEGG metabolic pathway impact analysis. The color and size of each node are based on the p values and pathway impact values, respectively. The “human Homo sapiens (KEGG)” library was selected to perform pathway enrichment analysis and topology analysis. The X-axis presents pathway impact values (based on Out-degree Centrality) and the Y-axis presents the respective p values (based on hypergeometric test). Each circle represents a pathway. The size of circle indicated the pathway impact score, whereas the color represents the p values. Larger circles represent higher impact factors, redder colors represent lower p values. Pathways with p values less than 0.05 were marked by name.

**Figure 6:**
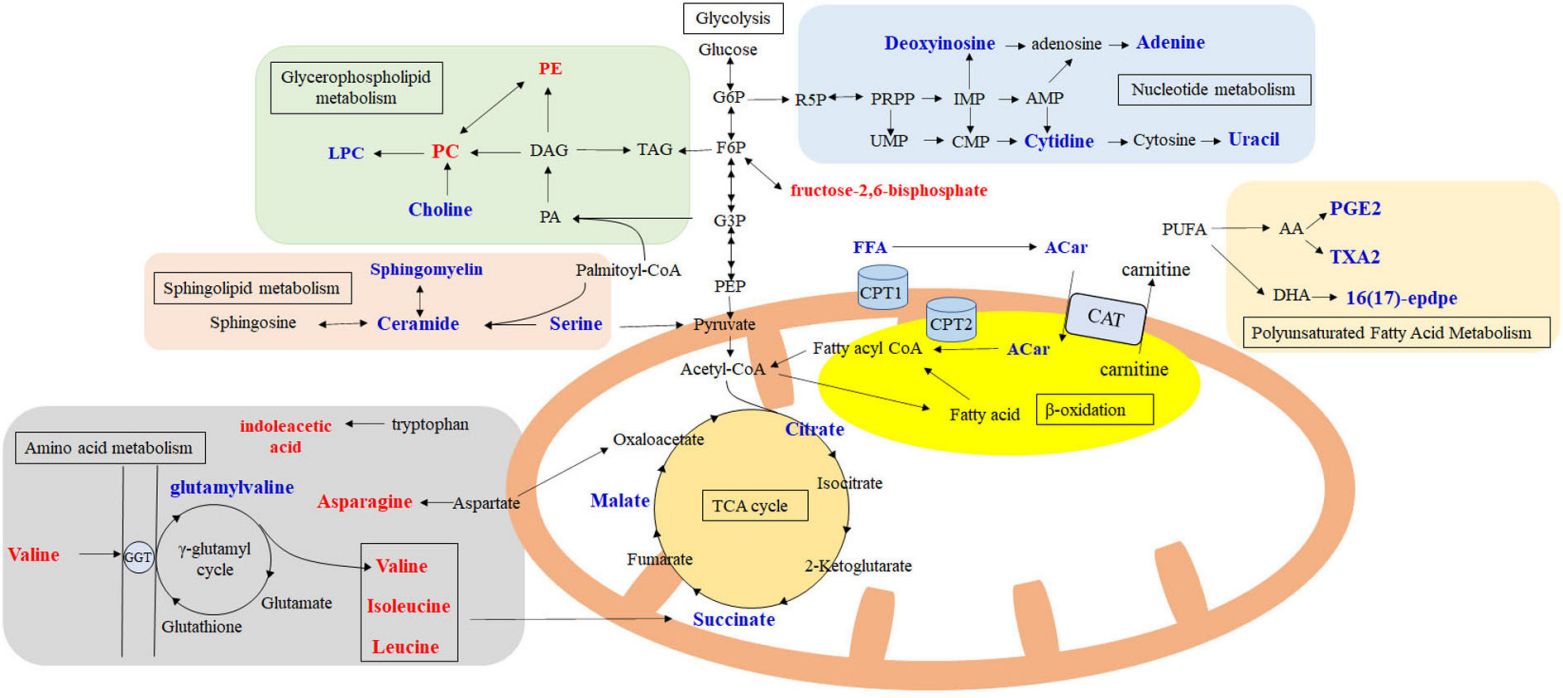
Altered of metabolites and metabolic pathways observed in patients with well response after dupilumab treatment. Differential metabolites are shaded in red or blue. Red represents evaluation after treatment and blue indicates a decrease after treatment. The average of all variants of lipids were calculated to represent the overall alteration of each subclass of lipid. Acar, Acylcarnitine; CAT, Carnitine Acytl Transferase; CPT1, carnitine palmitoyl transferase 1; CPT2, carnitine palmitoyl transferase 2; GGT, glutamyltransferase; FFA, Free fatty acid; LPC, Lysophophatidylcholine; PC, Phosphatidylcholine; PE, Phosphatidylethanolamine; PGE2, prostaglandins E2; TXA2, Thromboxane A2.

To further investigate the related metabolic pathways altered in patients with a good response to dupilumab, we utilized the metaboAnalyst 5.0 web server to perform pathway enrichment analysis. As shown in **Figure 5**, significantly different metabolites found to be enriched in six pathways at p < 0.05, including GP metabolism, valine, leucine, and isoleucine biosynthesis, the citrate cycle, arachidonic acid (AA) metabolism, pyrimidine metabolism, and SP metabolism. GP metabolism was the most significantly affected pathway after dupilumab treatment, based on pathway impact values and p values. We further constructed a metabolic network that showed clear changes in the levels of identified and annotated metabolites (based on the KEGG database) and related metabolic pathways (**Figure 6**).

## Discussion

In this study, we identified the global metabolic and lipidomic profiles of patients with AD before and after dupilumab treatment using LC-MS and GC-MS. Our findings suggest that the metabolic signature of patients with AD treated with dupilumab changed significantly after treatment, especially with respect to lipids. Most importantly, differential metabolites in patients with a good response to dupilumab therapy were found to be involved in GP metabolism, valine, leucine, and isoleucine biosynthesis, citrate cycle, AA metabolism, pyrimidine metabolism, and SP metabolism.

When integrating the differential metabolites screened from the metabolome and lipidome of all AD patients, we noticed that the most abundant classes were lipid and lipid-like molecules. Previous metabolomics and lipidomics studies have also shown that patients with AD have different lipid profiles than healthy individuals (14, 19). Therefore, we speculate that serum lipid metabolism may not only play an important role in the development of AD but also play a role in the biological response to treatment in patients with AD. Our results showed that lipid differential metabolites were dominated by PC, which are similar to many serum lipidomic studies on AD (19–21). However, these studies aimed to investigate the differential metabolic profile between patients with AD and healthy people, whereas in this study we included patients who received dupilumab for treatment to explore the change in the global metabolomics profile after therapy. Sakai et al. demonstrated that multiple variants of PC, including PC (32:1) and PC (36:4), were increased in patients with AD compared to healthy controls (19). Meanwhile, our findings suggest that PC (16:0/16:1) and PC (16:0/20:4), also known as PC (32:1) and PC (36:4), respectively, decreased after treatment (**Figure 2B**). This may indicate that dupilumab partially reverses the dysregulation of lipid metabolism in patients with AD.

The response rate of patients to dupilumab was 58% for EASI-75% at the end of the study period, which was higher than that reported in phase 3 clinical trials (EASI-75, 44%-51%) (22). We found that changes in metabolic profiles were larger in well-responders than in poor-responders. In addition, we found that the incidence of side effects was significantly higher in the well-responder group than in the poor-responder group. This suggests that metabolomic and lipidomic changes induced by dupilumab therapy may be due to reduction in skin inflammation and/or side effect. Subsequent pathway analysis of well responders’ metabolic profiles revealed that their differential metabolites were involved in six primary metabolic pathways, including GP metabolism, valine, leucine, and isoleucine biosynthesis, the citrate cycle, AA metabolism, pyrimidine metabolism, and SP metabolism.

To investigate the changes in the immunological cytokines associated with AD to the metabolomics and lipidomics, we first compared the profiles of Th1/Th2/Th17 cytokines in patients with AD before and after dupilumab treatment. In our study, we observed decline serum levels of IL-6, IL-10, and IFN-γ in AD patients after dupilumab therapy, which suggests dupilumab suppresses Th1/Th2 pathway. Our findings are partially consistent with previous studies (23). Notably, IFN-γ has not been reported previously to be decreased in the serum of AD patients receiving dupilumab. Next, the correlation analysis between metabolomics/lipidomics and immunological cytokines was further conducted. We found that many metabolites/lipids were associated with proinflammatory IL-4, IL-6 and anti-inflammatory IL-10 cytokine levels in pre-treatment samples. However, correlations between inflammatory cytokines and metabolites were poor in post-treatment samples. Together, these results indicate that the improvement of metabolic disorders by dupilumab may be independent of cytokines.

Among pathways affected by dupilumab therapy, GP metabolism was the most significantly altered metabolic pathway in this study. GPs are the main components of biological membranes. Increasing evidence shows that GP metabolism plays an important role in inflammation-associated skin diseases (24). We also found that for PCs, the most abundant class of lipids (25), several studies have confirmed their down-regulation in psoriasis patients (26, 27). Futhermore, Ottas et al. identified three PCs that were decreased in AD, including PC(38:5), PC(40:5), and PC(16:1/20:4). Another study conducted by Sakai et al. identified nine PCs that were increased in patients with AD, including PC(32:1), PC(34:1), PC(34:2), PC(36:0), PC(36:2), PC(36:4), PC(38:2), PC(38:3), PC(38:4) (10, 19). In the current study, patients displayed higher serum levels of 36 PCs and decreased levels of 10 PCs after treatment. Four variants of decreased PCs were consistent with the study by Sakai et al.: PC (14:0/18:1), PC (20:1/18:1), PC (22:4/16:0), and PC (22:4/14:0) (19). These variants of PC were found to be up-regulated in patients with AD in Sakai’s study, whereas our research showed that they were down-regulated after dupilumab treatment. In contrast, a previous study suggested that PC significantly decreased after omalizumab therapy, and high serum levels of PC have been reported as a factor related to the positive effect of omalizumab in AD (12). PC is synthesized by CDP-choline and hydrolyzed into lysophosphatidylcholine (LPC) and fatty acids by phospholipase A2 (PLA2) (28). High levels of PC may stimulate PLA2, which releases phospholipids for AA synthesis (29). According to our analysis, AA metabolism was one of the pathways identified to be significantly enriched following dupilumab treatment based on differential metabolites. Prostaglandin E2 (PGE2) and thromboxane A2 (TXA2) derived from AA contribute to the development of inflammation. A combined lipidomics and transcriptomics analysis performed by Töröcsik et al. suggested that AA and PGE2 were increased in the skin as well as in the serum of AD patients (30). Increased concentrations of PC and total PE and decreased concentrations of choline, LPC, FFA, PGE2, and TXA2 were observed after treatment in this study. Although PLA2 could not be directly detected, changes in metabolite levels in the GP and AA pathways may indicate that PLA2 activity was inhibited after treatment, thereby further reducing inflammation. We found that dupilumab alters GP metabolism in patients with AD, and have identified positive impacts attribute to these alterations. However, in some instances, increased levels of PC may be correlated with cancer risk (31). Further studies are required to confirm the effects of long-term dupilumab use on GP metabolism, which will provide important insights relevant to the clinical application of dupilumab.

Valine, leucine, and isoleucine, collectively referred to as branched-chain amino acids (BCAAs), are essential amino acids acquired solely from the diet. BCAAs affect many physiological processes such as energy homeostasis, inflammation, and glycolysis (32). Currently, researches on BCAAs are mainly focused on metabolic diseases wherein elevated levels of BCAAs have been reported to be correlated with type 2 diabetes and obesity (33). Few studies have evaluated the association between BCAAs and AD. In this study, AD patients exhibited higher serum levels of BCAAs after treatment than before treatment. The increase in BCAAs levels may arise from impaired catabolism of amino acids after excluding dietary factors. However, the mechanisms underlying this change remain unclear. Chronic BCAAs elevation is known to prevent the entry of aromatic amino acids into the brain, which may drive hunger (34), and this may explain why some patients complained of increased appetite post-treatment during follow-up. Notably, we found that the tryptophan derivative indole-acetic acid (IAA) increased after dupilumab treatment. Previously, Yu et al. found that the level of indole-3-aldehyde (a tryptophan derivative of the skin microbiota) in the skin of AD patients was significantly lower compared to healthy controls and demonstrated that topical application of indole derivatives in mouse models of AD can significantly alleviate skin inflammation (35). In addition, research has suggested that IAA can scavenge free radicals, inhibit oxidative stress, and reduce the production of proinflammatory cytokines (36). These results indicated that dupilumab may increase levels of indole derivatives, thereby reducing skin inflammation. However, further studies are needed to explore whether dupilumab affects the skin microbiome and its derivatives.

The tricarboxylic acid (TCA) cycle, also known as the citrate cycle, is considered the central hub of metabolic pathways (37). In eukaryotes, the TCA cycle only occurs in the matrix of the mitochondria, and the intermediates of the TCA cycle are retained within the mitochondria (38). Many researchers have suggested that under cellular stress conditions, TCA cycle intermediates such as citrate, itaconate, succinate, fumarate, and L-malate would accumulate and can cause cellular deficits. In asthma patients, studies have reported increased TCA intermediate concentrations (39, 40). In this study of AD patients, our results obtained from serum revealed that intermediates of the TCA cycle were reduced after dupilumab treatment, including citrate, succinate, and malate. Fructose 2,6 diphosphate, however, an agonist of key enzymes in glycolysis, was elevated. These findings also indicated that dupilumab therapy may attenuate oxidative stress-mediated inflammation and promote energy metabolism. A previous targeted metabolomics study conducted by Ottas et al. confirmed that the energy altered in patients with AD was evident by dysregulated acylcarnitines and phospholipids (10). Furthermore, increasing evidence has shown that metabolites of the TCA cycle can alter immune system responses. For example, prior studies have shown that citrate and succinate exert pro-inflammatory effects (41). The treatment of AD with dupilumab may reverse this pathological process by affecting the concentrations of these metabolites. However, the immunological effects of other intermediates are less studied and warrant further investigation.

Our results further provide evidence that alterations in pyrimidine metabolism are involved in responses to dupilumab. We identified several novel metabolites that had not previously been reported to be altered in AD that were reduced after treatment, including cytidine, uracil, deoxyinosine, and adenine. In contrast, although SP metabolism has been widely studied on stratum corneum of AD patients (42), limited data are available on serum levels of different Cer and SM species in AD. In the present study, we observed that the levels of SM decreased after treatment, whereas 24 species of Cer decreased, and eight species were elevated. A previous study found that high levels of serum SM were associated with the development of obesity and insulin resistance (43). However, the mechanisms underlying these findings have not yet been elucidated.

Some limitations to this study that may impact the interpretation of the findings. Firstly, the sample size was relatively small and there was a lack of an external validation cohort. Secondly, only one biological sample (serum) was used; more biological samples (urine and skin) would provide better insight into the comprehensive metabolic profile changes that occur in response to dupilumab treatment. Thirdly, this is a self-control study; although attempts were made to control for the influence of confounding factors, such as diet, exercise, and other treatment modalities, there may still be some confounding factors caused by differing exposures and timeframes that could affect the results. Further studies with large-scale cohorts using targeted approaches are needed to confirm these findings.

## Conclusions

In summary, we used LC-MS and GC-MS to characterize the global metabolic profile response to dupilumab therapy in patients with AD. OPLS-DA models were used to identify discriminatory features between poor and well responders. A metabolic signature of patients with positive responses to dupilumab therapy was subsequently established. These data demonstrate that dupilumab therapy may reverse AD pathophysiology and cause side effects by affecting the concentrations of specific metabolites and altering their associated metabolic pathways, including GP metabolism, valine, leucine, and isoleucine biosynthesis, citrate cycle, AA metabolism, pyrimidine metabolism, and SP metabolism.

## Data availability statement

The data presented in the study are deposited in the NIH Common Fund's National Metabolomics Data Repository, accession number IDST002302.

## Ethics statement

The studies involving human participants were reviewed and approved by the Regional Ethical Review Board of the Peking Union Medical College Hospital. The patients/participants provided their written informed consent to participate in this study.

## Author contributions

YZ and JS designed and supervised the study. LZ, XW, YH, YY, and WS performed experiments and analyzed the data. LZ contributed to sample collection. All authors contributed to the article and approved the submitted version.

## Acknowledgments

We thank Ruiqi Wang and Guodong Fu (Peking Union Medical College Hospital) for help in sample collection. The study has been supported by the National Natural Science Foundation of China (NO. 81971515); CAMS Innovation Fund for Medical Science (2021-I2M-1-017).

## Conflict of interest

The authors declare that the research was conducted in the absence of any commercial or financial relationships that could be construed as a potential conflict of interest.

## Publisher’s note

All claims expressed in this article are solely those of the authors and do not necessarily represent those of their affiliated organizations, or those of the publisher, the editors and the reviewers. Any product that may be evaluated in this article, or claim that may be made by its manufacturer, is not guaranteed or endorsed by the publisher.
