# Supplementary material for: Integrated metabolomics and lipidomics study of patients with atopic dermatitis in response to dupilumab
Source: Front Immunol. 2022 Oct 20;13:1002536. doi: 10.3389/fimmu.2022.1002536 (PMC9632449; doi:10.3389/fimmu.2022.1002536)
Supplement: Supplementary file 1 [file DataSheet_1.docx]

**1 Supplementary Data**

**GC-TOF-MS Analysis**

Samples (50μl) in Eppendorf (EP) tubes were extracted using 205 μL of precooled methanol extract (including internal L-2-Chlorophenylalanine, 1 mg/mL stock), followed by vortex mixing for 30 s and ultrasound for 10 min. After centrifugation at 4℃ for 15 min at 12000 rpm, we carefully transferred the 180 μL supernatant into a new 1.5-mL EP tube. We then took a 50-μL aliquot from each sample and mixed them with a quality control (QC) sample. The extract was then dried in a vacuum concentrator without heating. Next, 30 μL of methoxyamination hydrochloride (20 mg/mL in pyridine) was added and incubated at 80℃ for 30 min. Subsequently, we added 40 μL of BSTFA regent (1% TMCS, v/v) and incubated at 70℃ for 1.5 h. We then added 5 μL of fatty acid methyl ester (FAME) (in chloroform) to the QC sample after the samples cooled to room temperature. All samples were analyzed using GC coupled with time-of-flight (TOF)-MS (GC-TOF-MS).

GC-TOF-MS analysis was performed using an Agilent 7890 gas chromatograph coupled with a time-of-flight mass spectrometer. The system utilized a DB-5MS capillary column. 1 μL aliquot of sample was injected in splitless mode. Helium was used as the carrier gas, the front inlet purge flow was 3mL min−1, and the gas flow rate through the column was 1mL min−1. The initial temperature was kept at 50°C for 1min, then raised to 310 °C at a rate of 20°C min−1, then kept for 6min at 310°C. The injection, transfer line, and ion source temperatures were 280, 280and 250°C, respectively. The energy was -70eV in electron impact mode. The mass spectrometry data were acquired in full-scan mode with the m/z range of 50-500 at a rate of 12.5 spectra per second after a solvent delay of 4.83min.

**LC-MS Analysis**

Firsty, we describe the extraction of the metabolites. Samples (100 μL) were placed into an EP tube and extracted with 400 μL extract solution (acetonitrile:methanol = 1:1, containing an isotopically labelled internal standard mixture), followed by vortexing for 30 s, sonicatation for 10 min in an ice-water bath, and incubated for 1 h at -40℃ to precipitate proteins. The samples were then centrifuged at 12000 rpm for 15 min at 4℃. The supernatant was collected and transferred to a new glass vial for further analysis. The QC samples were then mixed from each sample.

Secondly, 480 μL extract solution (methyl tert-butyl ether (MTBE):methanol = 5:1) was added to 100 μL of the sample and vortexed for 30 s. After sonication for 10 min in an ice-water bath, samples were incubated at -40℃ for 1 h, then centrifuged at 3000 rpm for 15 min at 4℃. The supernatant was then placed in a fresh tube and dried in a vacuum concentrator at room temperature. The samples were reconstituted with 100 μL solution (dichloromethane:methanol = 1:1). After vortexing for 30 s, the samples were sonicated for 10 min in ice-water bath and then centrifuged at 13000 rpm for 15 min at 4 ℃. The supernatant was then transferred to a fresh glass vial for further analysis.

For metabolomics, LC-MS/MS analyses were performed using an UHPLC system (Vanquish, Thermo Fisher Scientific) with a UPLC BEH Amide column (2.1 mm × 100 mm, 1.7 μm) coupled to Q Exactive HFX mass spectrometer (Orbitrap MS, Thermo). The mobile phase consisted of 25 mmol/L ammonium acetate and 25 ammonia hydroxide in water（pH = 9.75）(A) and acetonitrile (B). The auto-sampler temperature was 4 ℃, and the injection volume was 2 μL. The QE HFX mass spectrometer was used for its ability to acquire MS/MS spectra on information-dependent acquisition (IDA) mode in the control of the acquisition software (Xcalibur, Thermo). In this mode, the acquisition software continuously evaluates the full scan MS spectrum. The ESI source conditions were set as following: sheath gas flow rate as 30 Arb, Aux gas flow rate as 25 Arb, capillary temperature 350 ℃, full MS resolution as 120000, MS/MS resolution as 7500, collision energy as 10/30/60 in NCE mode, spray Voltage as 3.6 kV (positive) or -3.2 kV (negative), respectively.

LC-MS/MS analyses were performed using an UHPLC system (1290, Agilent Technologies), equipped with a Kinetex C18 column (2.1 x 100 mm, 1.7 μm, Phenomen). The mobile phase A consisted of 40 % water, 60 % acetonitrile, and 10 mmol/L ammonium formate. The mobile phase B consisted of 10 % acetonitrile and 90 % isopropanol, which was added with 50 mL 10 mmol/L ammonium formate for every 1000 mL mixed solvent. The analysis was carried with elution gradient as follows: 0~1.0 min,40 % B;1.0~12.0 min,40 %~100 % B; 12.0~13.5 min, 100 % B; 13.5~13.7 min, 100 %~40 % B; 13.7~18.0 min, 40 % B. The column temperature was 55 ℃. The auto-sampler temperature was 4 ℃, and the injection volume was 2 μL (pos) or 2 μL (neg), respectively. The QE mass spectrometer was used for its ability to acquire MS/MS spectra on data- dependent acquisition (DDA) mode in the control of the acquisition software (Xcalibur 4.0.27, Thermo). In this mode, the acquisition software continuously evaluates the full scan MS spectrum. The ESI source conditions were set as following: sheath gas flow rate as 30 Arb, Aux gas flow rate as 10 Arb, capillary temperature 320 ℃(positive) ,300 ℃ (negative), full MS resolution as 70000, MS/MS resolution as 17500, collision energy as 15/30/45 in NCE mode, spray Voltage as 5 kV (positive) or -4.5 kV (negative), respectively.

**2 Supplementary Tables**

**Table S1 Criteria for inclusion and exclusion**

| criteria | detail |
| --- | --- |
| Inclusion criteria | 1. Age ≥ 18 years of age |
|  | 1. Dermatologist diagnosis of moderate to severe AD, EASI≥16 at baseline |
|  | 1. Eligible to receive dupilumab therapy for AD in accordance with the guidelines. Patients who are eligible were treated with a fixed schedule of 300mg dupilumab in 2-week intervals. Patients who did not achieve 16-week therapy were excluded. |
|  | 1. During the whole treatment process, the requirements for diet and exercise are roughly the same as before treatment, so as to keep the body healthy and balanced |
|  | 1. A 30-day washout period of systemic medications preceded treatment |
| Exclusion criteria | 1. Evidence of other skin diseases except for AD at baseline |
|  | 1. Pregnancy or breast feeding, |
|  | 1. Patients with permanent severe diseases, especially those affecting the immune system, except asthma |
|  | 1. Patients with severe mental illness |
|  | 1. Evidence of chronic metabolic disease, including Obesity, diabetes, fatty liver, osteoporosis, atherosclerotic cardiovascular and cerebrovascular diseases, and metabolic-related cancers (breast, colorectal, pancreatic, colon, and prostate cancer). |
|  | 1. Application of other systemic medications during treatment |

**Table S2 Total significantly different metabolites and lipids between before and after treatment group (VIP＞1 and p＜0.05)**

| Name | VIP | P | FC |
| --- | --- | --- | --- |
| 24,25-dihydrolanosterol | 2.43225497 | 0.00426943 | 3.35605736 |
| 5-methylcytidine | 2.98995506 | 7.9361E-05 | 2.1298301 |
| galactose | 1.96270171 | 0.03514491 | 2.04649138 |
| phenylalanyl-isoleucine | 3.22649408 | 0.00015245 | 1.70551587 |
| norvaline | 1.87175351 | 0.01534051 | 1.57624267 |
| 3-(4-hydroxyphenyl)-1-propano | 2.33735125 | 0.00269779 | 1.54941617 |
| 5-hydroxyindoleacetic acid | 2.79432442 | 0.00153238 | 1.50267305 |
| hydroxyurea | 1.47986465 | 0.01668196 | 1.41815385 |
| 6-methyl-3,5-heptadien-2-one | 2.44294877 | 0.00344194 | 1.34950327 |
| lactic acid | 1.55706474 | 0.00871168 | 1.28644567 |
| norophthalmic acid | 1.67235302 | 0.02240344 | 1.26984246 |
| alanine | 1.25692976 | 0.04583696 | 1.26579728 |
| 4-hydroxybenzaldehyde | 2.09077993 | 0.00387544 | 1.24731686 |
| uridine | 1.24780654 | 0.04128129 | 1.19486514 |
| 3,7,12-trihydroxycoprostane | 1.85205922 | 0.00918881 | 1.1727534 |
| 1-methyl-1,3-cyclohexadiene | 2.35555406 | 0.00470812 | 1.13971047 |
| serine | 1.44554605 | 0.04953236 | 1.06803142 |
| N-ethylglycine | 1.71038648 | 0.04818736 | 0.94183473 |
| glycine | 1.36904711 | 0.03780736 | 0.91604651 |
| uracil | 1.80656107 | 0.00964421 | 0.88108667 |
| 5,6-dihydrouracil | 2.07767629 | 0.00269463 | 0.87376743 |
| 4-hydroxy-2-butenoic acid gamma-lactone | 2.32182617 | 0.0043672 | 0.86602956 |
| 2,5-dihydro-2,4-dimethyloxazole | 1.32470822 | 0.00979776 | 0.85883169 |
| phenylpyruvic acid | 1.6778069 | 0.03038438 | 0.84323694 |
| N-methyl-alanine | 1.06963839 | 0.02105049 | 0.83849778 |
| 16-hydroxy hexadecanoic acid | 2.82946697 | 0.00388537 | 0.82664454 |
| 2-methyl-5-(1-propenyl) pyrazine | 1.70410822 | 0.04276142 | 0.81600853 |
| dibutyl phthalate | 1.71114239 | 0.03959594 | 0.80824396 |
| choline | 1.01697162 | 0.04218551 | 0.80412625 |
| malic acid | 1.69070308 | 0.02396001 | 0.80089494 |
| pyrrolidonecarboxylic acid | 1.46000435 | 0.02070462 | 0.79989649 |
| adenine | 1.88992589 | 0.01456768 | 0.79792072 |
| 9-decenoic acid | 2.25438416 | 0.0316924 | 0.79396414 |
| trans-hexadec-2-enoyl carnitine | 2.65974046 | 0.01206115 | 0.76242732 |
| hexanoylcarnitine | 2.24501809 | 0.00804725 | 0.755118 |
| 3,4-dihydroxybenzaldehyde | 2.52906725 | 0.04640714 | 0.72494465 |
| uracil-5-carboxylic acid | 1.42515268 | 0.04129873 | 0.65696898 |
| glutamylvaline | 1.58677649 | 0.02694978 | 0.65242112 |
| succinic acid | 3.01569817 | 0.0003231 | 0.44221147 |
| deoxyinosine | 1.71088883 | 0.01639028 | 0.3648292 |
| 3-hydroxypyridine | 4.28701189 | 3.1756E-11 | 0.22921322 |
| PC(18:4/18:5) | 2.67728724 | 1.9344E-07 | 4.5020723 |
| PC(2:0/16:2) | 1.28839514 | 0.02995039 | 4.00174773 |
| OxPC(18:1/18:1+3O) | 2.39984308 | 2.9882E-07 | 2.83059658 |
| PC(20:4/20:4) | 3.34016176 | 3.3062E-06 | 2.80305969 |
| LPC(22:5) | 2.71825424 | 4.4911E-06 | 2.35925569 |
| LPC(22:6) | 3.03838324 | 1.9122E-07 | 2.22411851 |
| PC(20:5e/12:0) | 2.20433529 | 3.5453E-05 | 2.18356821 |
| PC(22:2/18:5) | 2.90720119 | 4.0651E-06 | 2.1396453 |
| PC(22:4/18:5) | 3.26946888 | 6.7786E-11 | 2.10794961 |
| PI(2:0/23:0) | 2.35957364 | 1.9269E-06 | 2.10246055 |
| OxPC(16:0/18:2+2O) | 3.02881962 | 2.6981E-09 | 2.08418274 |
| TAG(12:2/19:5/19:5) | 3.06920507 | 3.1966E-08 | 2.06593907 |
| PC(2:0/13:0) | 1.97667149 | 0.00011133 | 2.03104979 |
| PC(16:1/22:6) | 3.01652501 | 5.3865E-09 | 2.01648863 |
| PI(20:3/20:3) | 3.36429528 | 4.2041E-12 | 2.00068627 |
| PC(20:1/16:4) | 2.33782626 | 0.0001186 | 2.00060626 |
| TAG(12:1/19:5/19:5) | 3.57721143 | 5.2733E-14 | 1.99922986 |
| PC(3:0/18:4) | 1.96977635 | 9.7739E-06 | 1.99042494 |
| SHexCer(d30:1) | 3.67906291 | 2.1662E-16 | 1.98343225 |
| PC(22:1/18:4) | 3.48859729 | 1.0266E-12 | 1.9705623 |
| PC(16:3/26:4) | 2.86644684 | 2.1968E-08 | 1.95472045 |
| PC(18:5e/8:0) | 1.87065616 | 1.6857E-05 | 1.9521461 |
| OxPI(18:0/20:4+3O(2Cyc)) | 3.23023318 | 4.392E-11 | 1.93567922 |
| PC(4:0/5:0) | 2.71293654 | 5.6797E-07 | 1.92632361 |
| PC(16:0/20:5) | 2.37634875 | 5.9157E-05 | 1.89619179 |
| PC(24:4/18:5) | 2.60654107 | 2.0479E-07 | 1.89564968 |
| PC(16:2/16:2) | 2.05836233 | 0.00032302 | 1.8095865 |
| PC(18:3/22:6) | 2.06143499 | 6.4766E-05 | 1.80588879 |
| PC(16:2/18:5) | 2.34115591 | 1.9443E-05 | 1.79166559 |
| PC(20:5/20:5) | 1.03678253 | 0.03630308 | 1.78953717 |
| PC(18:2/18:3) | 2.26020267 | 4.3128E-05 | 1.77775496 |
| SHexCer(d33:2) | 2.62624398 | 2.467E-05 | 1.77580933 |
| SQDG(16:1/22:4) | 2.87568978 | 2.8534E-08 | 1.74479129 |
| SHexCer(d28:1) | 3.05813221 | 4.5633E-08 | 1.71795167 |
| PC(22:6/7:0) | 2.73933894 | 7.1306E-07 | 1.71185573 |
| PC(22:6/6:0) | 2.43161046 | 0.00036303 | 1.71171716 |
| OxPE(16:0/20:5+1O(1Cyc)) | 2.52210788 | 6.0879E-05 | 1.70875226 |
| SQDG(20:0/20:0) | 2.73498414 | 3.8511E-07 | 1.70286704 |
| PC(20:3/22:6) | 2.68852477 | 6.3289E-06 | 1.69792639 |
| SHexCer(d27:1) | 2.60839846 | 3.3895E-05 | 1.69791373 |
| PC(22:6/8:0) | 2.23963936 | 0.00072268 | 1.69333541 |
| PC(18:1/22:6) | 2.6939882 | 1.0806E-05 | 1.67425722 |
| GlcADG(18:1/24:2) | 2.83770133 | 5.1612E-08 | 1.67375827 |
| PC(5:0/12:0) | 1.34281303 | 4.6371E-05 | 1.67366604 |
| DGTS(6:0/22:5) | 1.21316867 | 0.01735346 | 1.66781843 |
| GlcADG(14:0/22:3) | 3.14278374 | 5.5094E-10 | 1.6586402 |
| PC(2:0/17:1) | 2.27200084 | 1.6439E-06 | 1.63892095 |
| PC(18:4/24:4) | 1.77781184 | 0.00773793 | 1.62790496 |
| LPC(20:5) | 2.24302455 | 7.3314E-05 | 1.62412457 |
| PC(20:1/18:4) | 2.95790536 | 5.182E-08 | 1.61388966 |
| PC(20:4/22:4) | 2.43685497 | 2.9882E-06 | 1.61252607 |
| PE(22:5/10:0) | 2.19199446 | 0.00054895 | 1.60992592 |
| PC(18:4/16:4) | 2.62965091 | 5.7806E-06 | 1.60924388 |
| PC(2:0/17:2) | 1.40891421 | 5.7835E-05 | 1.6090069 |
| PC(20:5/10:0) | 1.8431155 | 0.00028618 | 1.60092872 |
| PC(2:0/21:1) | 3.00424737 | 2.8703E-09 | 1.59631831 |
| SHexCer(d29:1) | 2.5707416 | 3.227E-05 | 1.59282913 |
| OxPC(16:0/18:1+3O) | 2.17881068 | 1.1732E-06 | 1.58273794 |
| PC(18:5/13:1) | 2.48857831 | 0.00041016 | 1.57709383 |
| PC(22:6/4:0) | 2.59670037 | 1.5349E-06 | 1.57225614 |
| TAG(12:0/18:5/18:5) | 3.17124997 | 3.7911E-10 | 1.57111276 |
| PC(9:0/22:6) | 1.94751137 | 0.00057667 | 1.56959639 |
| OxPI(18:0/22:6+3O) | 2.27946245 | 8.1189E-06 | 1.56834957 |
| TAG(12:0/19:5/19:5) | 3.1955411 | 1.572E-09 | 1.5588298 |
| SQDG(12:0/16:4) | 2.25576405 | 0.00016106 | 1.55305662 |
| PC(22:6/3:0) | 2.36956533 | 2.8785E-05 | 1.54998754 |
| ACar(24:2) | 1.94283132 | 0.00124994 | 1.53781061 |
| PC(14:1/16:4) | 2.69300635 | 8.603E-06 | 1.52947658 |
| TAG(12:2/12:2/13:1) | 1.1598449 | 0.01450247 | 1.51984087 |
| PC(22:6e/5:0) | 2.31784029 | 0.00018374 | 1.51552325 |
| PC(2:0/7:0) | 2.19076262 | 6.5192E-05 | 1.51213676 |
| PC(13:0/22:5) | 2.71742241 | 7.6236E-09 | 1.50773057 |
| PC(2:0/22:1) | 1.45268472 | 0.00479055 | 1.50483454 |
| OxPC(18:0/18:2+2O) | 2.318122 | 0.00015258 | 1.5043467 |
| OxPI(18:0/20:3+2O(1Cyc)) | 2.15059287 | 0.00059311 | 1.49862428 |
| OxPC(16:0/20:3+2O) | 1.51298852 | 0.00870954 | 1.497701 |
| PC(18:5/10:0) | 2.43871223 | 4.162E-05 | 1.49416624 |
| PC(11:0/22:5) | 2.88851749 | 1.3166E-10 | 1.48810554 |
| PC(22:5/9:0) | 2.23301202 | 0.00011665 | 1.4874332 |
| SHexCer(d30:2) | 2.75612892 | 3.4222E-08 | 1.48246661 |
| PC(18:2/22:6) | 2.13420824 | 0.00453532 | 1.47171896 |
| PC(22:5/10:0) | 2.98145971 | 3.1568E-07 | 1.46767045 |
| GlcADG(14:1/24:2) | 2.3663797 | 7.7187E-06 | 1.46392601 |
| ACar(24:3) | 1.98119207 | 0.00184076 | 1.45843663 |
| TAG(12:1/14:0/14:1) | 1.13438709 | 0.01462505 | 1.45606138 |
| PC(22:5/13:1) | 1.72826963 | 1.4232E-05 | 1.44109903 |
| TAG(17:1/17:1/22:6) | 1.45334009 | 0.01053124 | 1.4381653 |
| PC(20:5/8:0) | 2.17273694 | 0.00015237 | 1.42661047 |
| PC(22:6/22:6) | 1.9509755 | 0.00722678 | 1.41209745 |
| LPA(18:0) | 1.74995864 | 0.02971766 | 1.39840431 |
| PC(12:0/22:6) | 2.41398145 | 2.3154E-05 | 1.39412512 |
| PC(16:3/16:3) | 2.31349523 | 5.3431E-05 | 1.39064061 |
| PC(2:0/19:0) | 2.14857572 | 0.00053739 | 1.3830513 |
| SHexCer(d27:2) | 1.55633804 | 0.00360032 | 1.38153614 |
| PC(16:0/22:6) | 2.02899804 | 0.02881349 | 1.38017852 |
| HexCer/NS(d21:3/16:0) | 1.10329935 | 0.04920906 | 1.37833946 |
| PC(6:0/15:0) | 2.46681296 | 3.8594E-05 | 1.37107144 |
| PC(20:5/9:0) | 1.81864954 | 0.02725985 | 1.36944739 |
| PC(22:5/5:0) | 2.32198253 | 2.5514E-05 | 1.36345021 |
| PE(18:2/20:2) | 1.13212151 | 0.03447408 | 1.36259851 |
| PE(22:5/6:0) | 1.8647576 | 0.00179016 | 1.36121509 |
| TAG(12:0/14:0/16:0) | 1.07504445 | 0.01099133 | 1.35961047 |
| PE(22:6/20:5) | 1.137475 | 0.03006413 | 1.35559262 |
| PC(18:5/15:1) | 1.96106738 | 0.00092844 | 1.35478313 |
| PC(2:0/21:0) | 2.12109393 | 0.00144688 | 1.33534943 |
| PC(6:0/22:6) | 1.82534922 | 0.00089456 | 1.3289409 |
| PC(22:5/6:0) | 2.11035963 | 0.00019942 | 1.31946516 |
| TAG(18:1/18:1/18:2) | 1.12857299 | 0.00368781 | 1.31500221 |
| PC(3:0/22:1) | 1.33811733 | 0.01853082 | 1.30786451 |
| PE(2:0/17:1) | 1.56551241 | 0.02396801 | 1.30742331 |
| PC(9:0/18:5) | 1.67685076 | 0.02349009 | 1.3066118 |
| PC(20:5/5:0) | 1.60653027 | 0.00977023 | 1.30621152 |
| PC(8:0/22:6) | 1.54120396 | 0.00489034 | 1.30612546 |
| SHexCer(d26:1) | 2.07296983 | 0.0003716 | 1.30250395 |
| PC(20:5/6:0) | 1.64612642 | 0.01070688 | 1.30152112 |
| PC(16:0/16:1) | 1.37192972 | 0.01150143 | 1.29737424 |
| DGTS(4:0/27:0) | 1.16453281 | 0.0352353 | 1.28953381 |
| ACar(22:2) | 1.78662017 | 0.01038627 | 1.28927227 |
| PC(5:0/18:0) | 1.52825485 | 0.0159014 | 1.27630169 |
| PC(18:5/14:1) | 1.68049556 | 0.00084423 | 1.27522038 |
| PC(5:0/22:6) | 1.20507433 | 0.03128098 | 1.26700587 |
| PC(18:3/18:5) | 1.23984493 | 0.02976458 | 1.25330378 |
| PC(12:0/12:0) | 1.65750135 | 0.02461005 | 1.24940534 |
| PC(18:1/22:6) | 1.66190803 | 0.0029007 | 1.24137781 |
| PC(4:0/26:4) | 2.13842757 | 5.7526E-05 | 1.2151115 |
| PC(22:5/22:5) | 1.15988976 | 0.02597376 | 1.19079471 |
| PI(18:0/18:1) | 1.23190814 | 0.02364481 | 1.1790473 |
| SHexCer(d37:0) | 1.04088199 | 0.04306388 | 1.12922532 |
| GlcADG(19:2/20:3) | 1.23427266 | 0.01899136 | 0.92029943 |
| PI(22:5/22:6) | 1.31262086 | 0.0404974 | 0.91154224 |
| PI(22:6/22:6) | 1.25050007 | 0.03239603 | 0.91027221 |
| TAG(18:3/22:7/22:7) | 1.38401483 | 0.04572201 | 0.90687112 |
| Cer/ADS(d14:0/26:1) | 1.23301527 | 0.0394688 | 0.88416855 |
| SM(d14:1/28:2) | 1.21560998 | 0.02414427 | 0.88307609 |
| PC(16:0/20:4) | 1.06509763 | 0.03100624 | 0.87664072 |
| TAG(13:1/13:1/13:1) | 1.02265103 | 0.02094041 | 0.85968851 |
| Cer/AS(d24:3/21:1) | 1.06735269 | 0.01476634 | 0.85700185 |
| ACar(24:1) | 1.06056947 | 0.04963787 | 0.85610048 |
| SQDG(13:0/24:4) | 1.16781273 | 0.00654957 | 0.85588319 |
| SQDG(12:0/19:1) | 1.30287503 | 0.00520961 | 0.85474948 |
| LPE(22:6) | 1.2143152 | 0.03332199 | 0.85233521 |
| SM(d14:1/30:2) | 1.07832549 | 0.02762294 | 0.85174679 |
| HexCer/NDS(d14:0/30:1) | 1.16816407 | 0.03849326 | 0.84715814 |
| GlcADG(20:0/20:0) | 1.11819807 | 0.03660708 | 0.84714333 |
| SQDG(17:2/22:6) | 1.64673369 | 0.00029628 | 0.84602237 |
| Cer/NDS(d21:0/23:1) | 1.0698642 | 0.03765136 | 0.84319155 |
| TAG(13:1/18:4/18:4) | 1.00480882 | 0.03652036 | 0.8421755 |
| Cer/EODS(d14:0/30:2/O/18:2) | 1.58984235 | 0.01377713 | 0.84208466 |
| Cer/AP(t17:1/26:0) | 1.3157199 | 0.03872522 | 0.8375184 |
| FAHFA(18:0/20:2) | 1.10317375 | 0.04558577 | 0.83689588 |
| HexCer/NS(d18:1/24:1) | 1.04712732 | 0.04841163 | 0.83363892 |
| Cer/ADS(d27:0/17:0) | 1.16976139 | 0.02289328 | 0.83065863 |
| MAG(16:0) | 1.20427981 | 0.00753669 | 0.83045848 |
| HBMP(12:0/12:0/22:4) | 1.08003184 | 0.04989595 | 0.829351 |
| Cer/AP(t19:1/25:0) | 1.28100693 | 0.0270713 | 0.82722952 |
| Cer/NS(d20:1/24:0) | 1.24315402 | 0.02054688 | 0.82611553 |
| GlcADG(21:2/26:4) | 1.11405721 | 0.01835614 | 0.8218334 |
| SM(d14:0/20:2) | 1.37630206 | 0.0081168 | 0.81615911 |
| Cer/AP(t19:1/26:0) | 1.302042 | 0.0116461 | 0.81599707 |
| Cer/AP(t14:1/29:1) | 1.57009584 | 0.01316991 | 0.81473308 |
| Cer/AP(t14:0/31:0) | 1.14422511 | 0.04039414 | 0.81399483 |
| TAG(14:3/14:3/22:0) | 1.28529842 | 0.03995988 | 0.81170754 |
| TAG(12:0/12:0/20:2) | 1.54614985 | 0.01011119 | 0.80992471 |
| SM(d14:1/24:2) | 1.05632352 | 0.04404588 | 0.80668592 |
| ACar(18:1) | 1.21164072 | 0.0079948 | 0.80657273 |
| FA(16:0) | 1.43397 | 0.01159388 | 0.80328864 |
| PG(18:1/18:2) | 1.04991183 | 0.0029341 | 0.80263139 |
| ACar(14:0) | 1.1306793 | 0.03118062 | 0.80238839 |
| PC(18:4e/22:6) | 1.07009732 | 0.0351774 | 0.79989463 |
| PI(27:0/22:6) | 1.18090353 | 0.01562133 | 0.79969153 |
| Cer/AP(t16:1/26:0) | 1.48597346 | 0.00723204 | 0.79642193 |
| TAG(12:0/12:0/20:1) | 1.79279348 | 0.00193043 | 0.79621164 |
| TAG(12:0/12:0/19:5) | 1.54516711 | 0.0004076 | 0.79550852 |
| LPA(16:0) | 1.2348004 | 0.02260038 | 0.79323602 |
| Cer/EODS(d14:0/24:0/O/18:1) | 1.10251146 | 0.02602153 | 0.79269194 |
| Cer/AP(t14:1/31:1) | 1.29441868 | 0.03198804 | 0.78467162 |
| Cer/EODS(d14:0/30:1/O/16:0) | 1.09045013 | 0.02358987 | 0.78200799 |
| HexCer/NS(d14:3/12:1) | 1.28061791 | 0.00841802 | 0.77733525 |
| SQDG(12:0/20:5) | 1.5026475 | 0.00115744 | 0.77380129 |
| LPE(18:0) | 1.69938797 | 0.0092318 | 0.77298924 |
| SM(d14:0/29:1) | 1.29604316 | 0.00832253 | 0.76318246 |
| Cer/ADS(d23:0/19:1) | 1.16903125 | 0.0331429 | 0.76103789 |
| ACar(17:1) | 1.36362655 | 0.01854879 | 0.75682146 |
| FA(20:1) | 1.28506133 | 0.01526049 | 0.75627538 |
| GlcADG(21:0/21:0) | 1.46228731 | 0.01059245 | 0.75609165 |
| SQDG(19:0/22:6) | 1.38409427 | 0.0017027 | 0.75511905 |
| ACar(16:1) | 1.39213749 | 0.01178591 | 0.75243566 |
| DAG(18:0/20:4) | 1.36957513 | 0.0197848 | 0.75181232 |
| Cer/AP(t14:0/30:0) | 1.19196527 | 0.02788742 | 0.75109893 |
| FA(17:1) | 1.21134566 | 0.04807106 | 0.74847114 |
| Cer/AP(t30:2/15:1) | 1.71814523 | 0.00660815 | 0.74815974 |
| PC(2:0/2:0) | 1.03890837 | 0.00166234 | 0.74556811 |
| FA(22:4) | 1.25816176 | 0.01350325 | 0.74515084 |
| ACar(20:1) | 1.16965986 | 0.02841742 | 0.74222722 |
| Cer/AP(t21:2/23:0) | 1.58251464 | 0.03033007 | 0.74033814 |
| Cer/AP(t14:0/32:1) | 1.55756352 | 0.0067259 | 0.73569327 |
| LPC(20:1) | 1.3567707 | 0.01498636 | 0.72800117 |
| SM(d14:0/28:2) | 1.51065072 | 0.01661646 | 0.72712819 |
| PI(9:0/22:3) | 1.46100572 | 0.00157324 | 0.71944278 |
| Cer/NS(d20:1/24:1) | 1.68239978 | 0.00191352 | 0.71850854 |
| FAHFA(18:1/20:3) | 1.42516414 | 0.00870218 | 0.69740522 |
| FAHFA(18:2/20:4) | 1.38149755 | 0.00474823 | 0.6949321 |
| PC(14:0/19:0) | 1.25384958 | 0.03804268 | 0.69427601 |
| FA(16:1) | 1.14734412 | 0.04509914 | 0.69352238 |
| ACar(19:1) | 1.25130021 | 0.01899889 | 0.69169489 |
| SM(d14:0/30:2) | 1.63312115 | 0.00343876 | 0.68832904 |
| DGTS(2:0/20:2) | 1.15047257 | 0.01979031 | 0.68675834 |
| DGTS(5:0/17:2) | 1.30181804 | 0.00488989 | 0.6736341 |
| FAHFA(18:1/18:0) | 1.28186344 | 0.02258368 | 0.66770247 |
| ACar(13:1) | 1.30384653 | 0.01452605 | 0.66274546 |
| PC(18:4/18:0) | 1.71361373 | 0.00046712 | 0.65962908 |
| FAHFA(18:1/22:3) | 1.42330787 | 0.00423014 | 0.6587245 |
| FAHFA(18:2/18:1) | 1.34281684 | 0.00610227 | 0.64866052 |
| Cer/NDS(d18:0/24:1) | 1.34379323 | 0.02978073 | 0.64475958 |
| SM(d14:0/26:1) | 1.38415629 | 0.03619282 | 0.61517016 |
| GlcADG(18:2/20:3) | 1.37209425 | 0.01656828 | 0.61424313 |
| FAHFA(20:4/20:3) | 1.71047527 | 6.266E-05 | 0.4799964 |
| TAG(12:1/12:1/18:2) | 2.04474487 | 0.00047234 | 0.44488946 |

**3 Supplementary Figures**

**
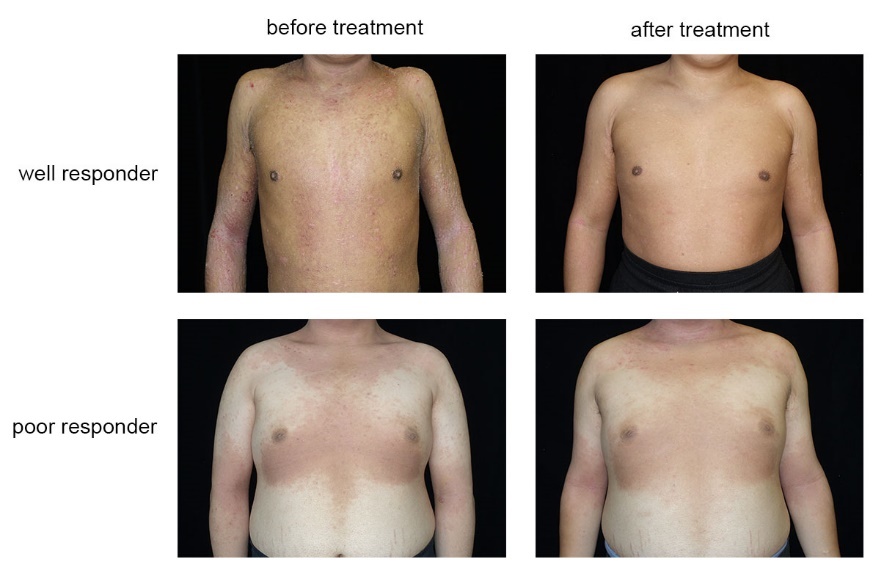
**

**Figure S1. Representative pictures of the responders and poor responders in patients with atopic dermatitis.**

**
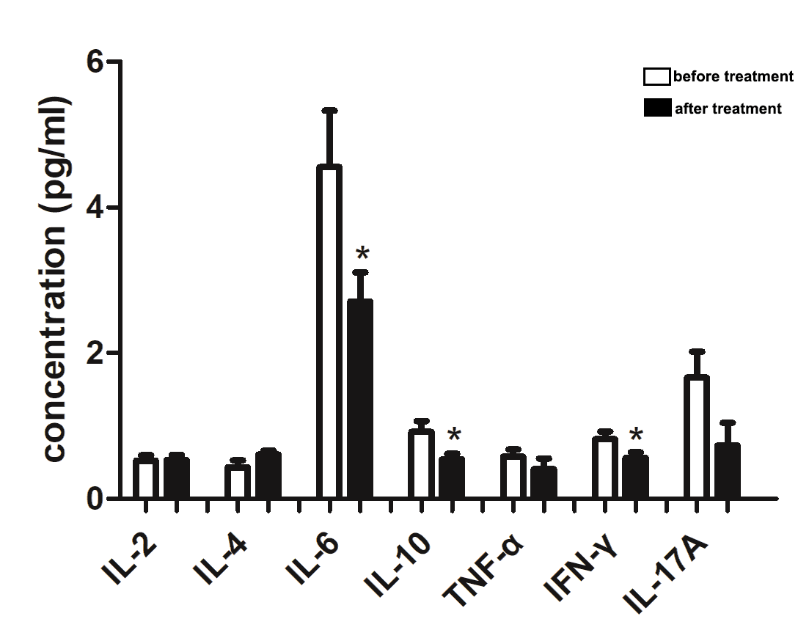
**

**Figure S2. Concentration of cytokines in serum samples of AD patients, prior and after dupilumab treatment.** Data are given as mean ± SEM, *Statistical significance.

**
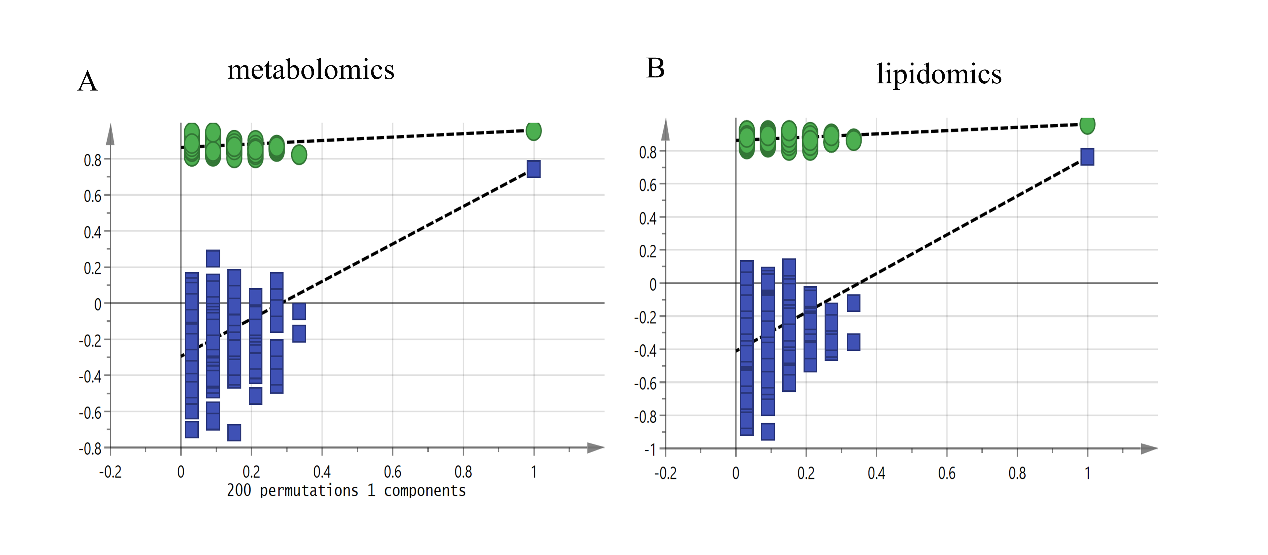
**

**Figure S3. OPLS-DA permutation plot of total patients.**


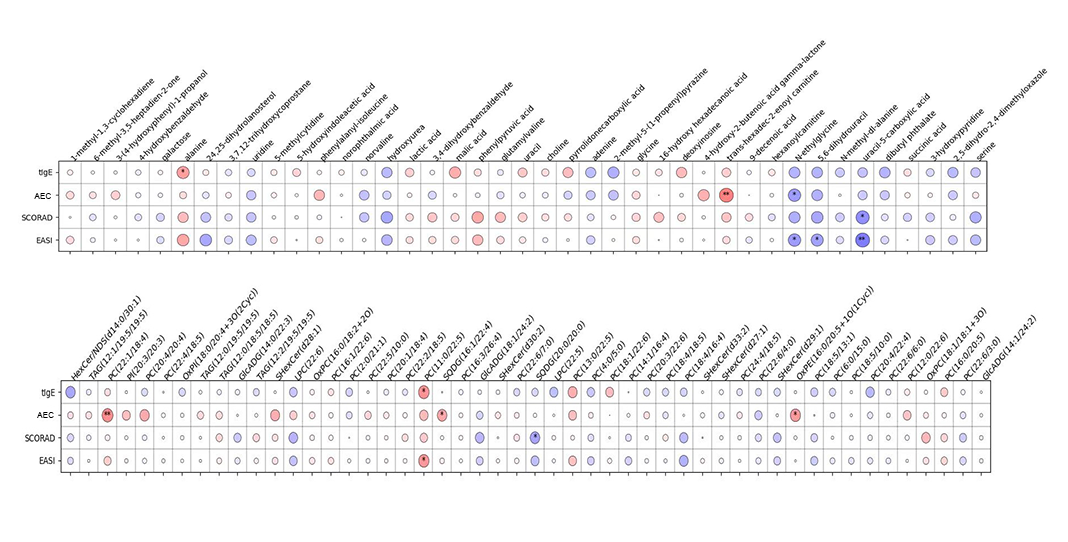


**Figure S4. Correlation between** **serum metabolites/lipids and clinical parameters before treatment.** Correlations were analyzed by Pearson’s correlation test. Color red to blue represents correlation coefficient. * represents P<0.05, ** represents P<0.01.

AEC, absolute eosinophil count; EASI, eczema area and severity index; HexCer/NDS, Hexosylceramide non-hydroxyfatty acid-dihydrosphingosine; TAG, triacylglycerol; PC, Phosphatidylcholine; OxPI, Oxidized phosphatidylinositol; GlcADG, glucuronosyldiacylglycerol; LPC, Lysophophatidylcholine; OxPE, Oxidized phosphatidylethanolamine; OxPC, Oxidized phosphatidylcholine; SCORAD, severity scoring of atopic dermatitis; SHexCer, SulfurHexosylceramide hydroxyfatty acid; SQDG, Sulfoquinovosyl diacylglycerol; tIgE, total serum IgE.

**
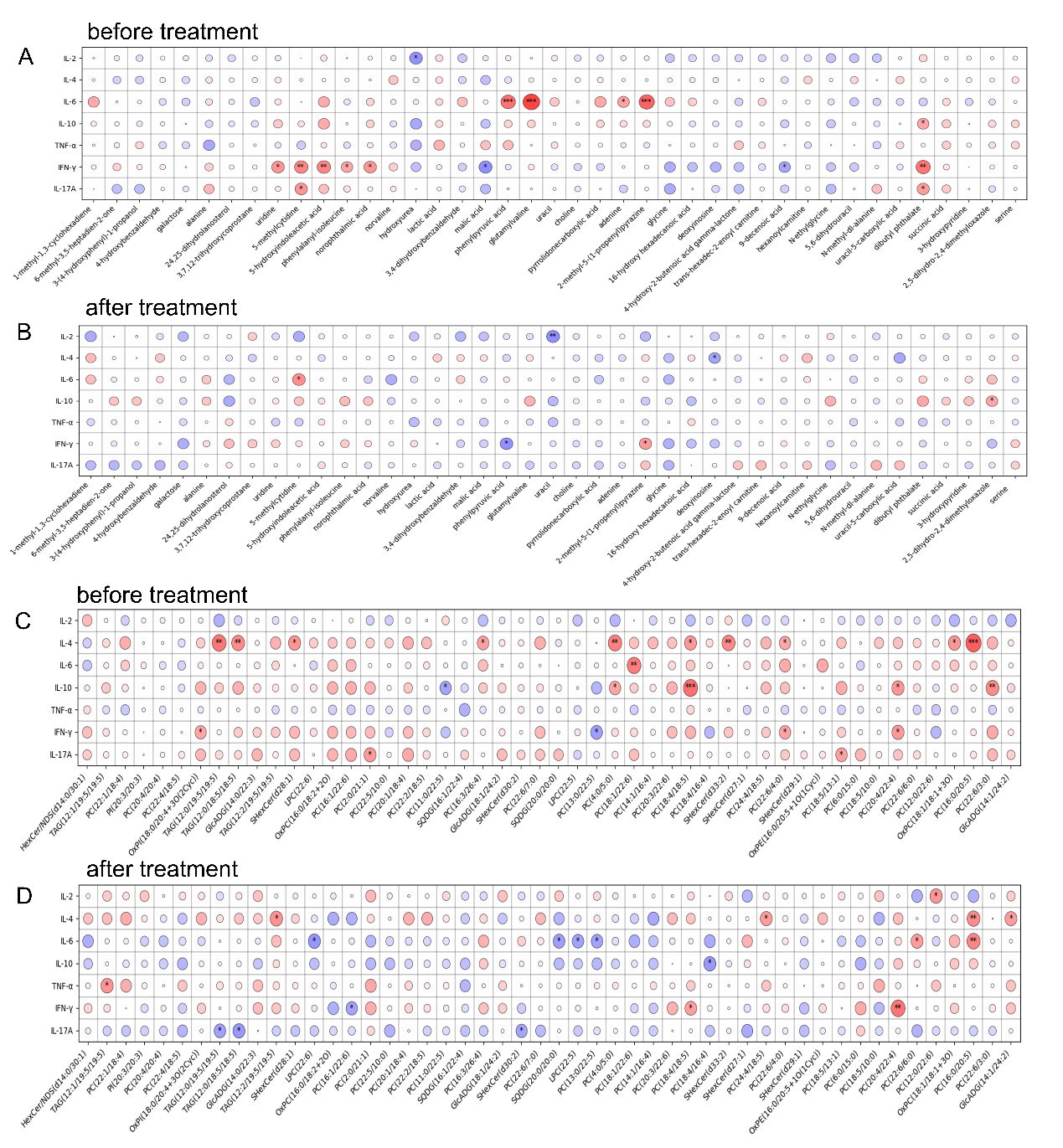
**

**Figure S5 Correlations between serum metabolites/lipids and the cytokine levels.**

Correlations between metabolites and cytokines in serum samples from patients before(A) and after receiving therapy (B). Correlations between top 50 lipids and cytokines in serum sample from patients before(C) and after receiving therapy (D). The Pearson correlation coefficient test was used to analyze correlations between metabolites/lipids and concentrations of cytokines. Color red to blue represents correlation coefficient. * represents P<0.05, ** represents P<0.01, *** represents P<0.001.

IL-2, interleukin-2; IL-4, Interleukin-4; IL-6, Interleukin-6; IL-10, Interleukin-10; TNF-α, tumor necrosis factor-α; IFN-γ, interferon-γ; IL-17A, Interleukin-17A; HexCer/NDS, Hexosylceramide non-hydroxyfatty acid-dihydrosphingosine, TAG, triacylglycerol; PC, Phosphatidylcholine; OxPI, Oxidized phosphatidylinositol; GlcADG, glucuronosyldiacylglycerol; LPC, Lysophophatidylcholine; OxPE, Oxidized phosphatidylethanolamine; OxPC, Oxidized phosphatidylcholine; SCORAD, severity scoring of atopic dermatitis; SHexCer, SulfurHexosylceramide hydroxyfatty acid; SQDG, Sulfoquinovosyl diacylglycerol.

**
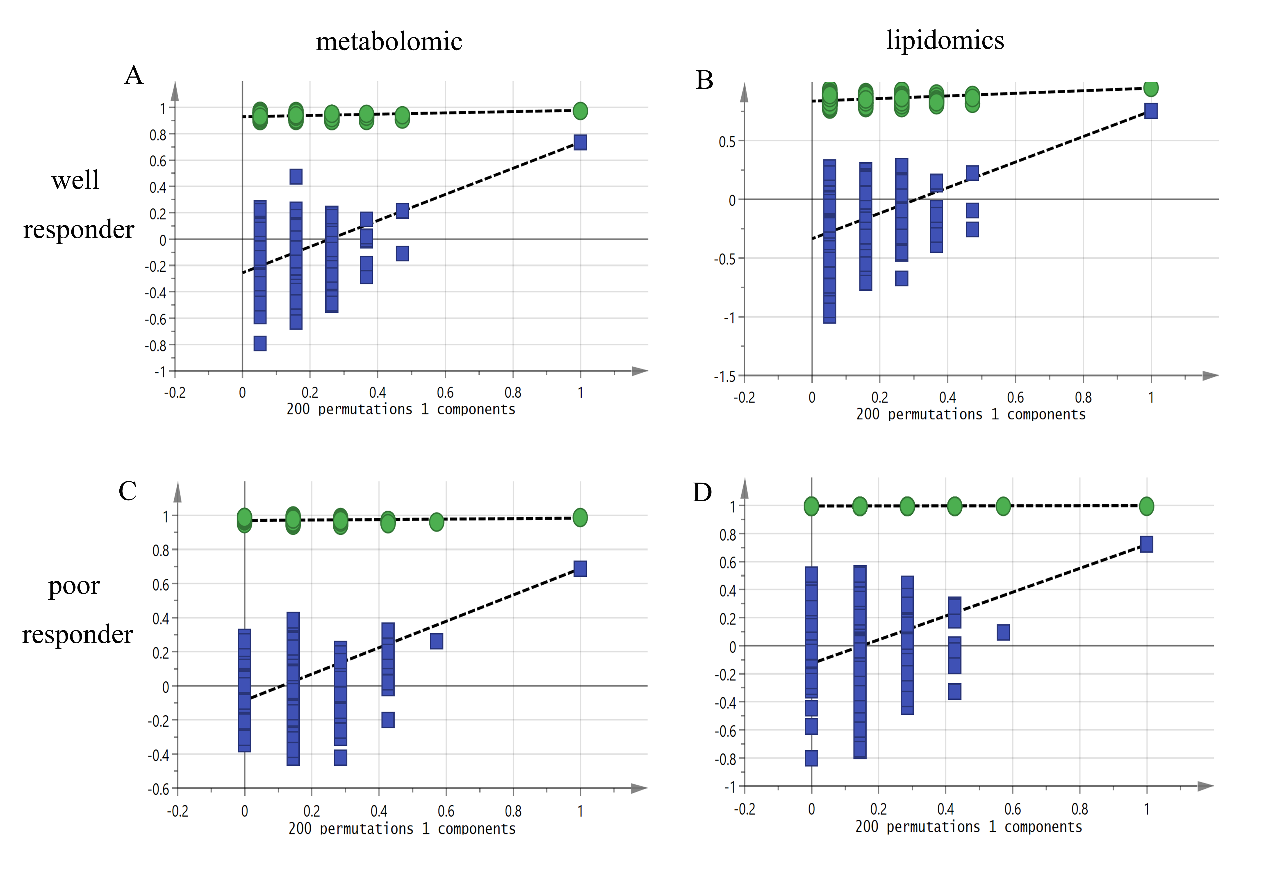
**

**Figure S6. OPLS-DA permutation plot of well responder and poor responder group.**
